# Investigating potential novel therapeutic targets and biomarkers for ankylosing spondylitis using plasma protein screening

**DOI:** 10.3389/fimmu.2024.1406041

**Published:** 2024-08-09

**Authors:** Wenkang You, Yanbin Lin, Mingzhong Liu, Zhangdian Lin, Rongjie Ye, Canhong Zhang, Rongdong Zeng

**Affiliations:** ^1^ Department of Orthopaedics, Quanzhou First Hospital Affiliated to Fujian Medical University, Quanzhou, China; ^2^ School of Medicine, Xiamen University, Xiamen, Fujian, China

**Keywords:** ankylosing spondylitis, Mendelian randomization, plasma protein, therapeutic targets, biomarkers

## Abstract

**Background:**

Ankylosing spondylitis (AS) is a chronic inflammatory disease affecting the spine and sacroiliac joints. Recent genetic studies suggest certain plasma proteins may play a causal role in AS development. This study aims to identify and characterize these proteins using Mendelian randomization (MR) and colocalization analyses.

**Methods:**

Plasma protein data were obtained from recent publications in *Nature Genetics*, integrating data from five previous GWAS datasets, including 738 cis-pQTLs for 734 plasma proteins. GWAS summary data for AS were sourced from IGAS and other European cohorts. MR analyses were conducted using “TwoSampleMR” to assess causal links between plasma protein levels and AS. Colocalization analysis was performed with the coloc R package to identify shared genetic variants. Sensitivity analyses and protein-protein interaction (PPI) network analyses were conducted to validate findings and explore therapeutic targets. We performed Phenome-wide association study (PheWAS) to examine the potential side effects of drug protein on AS treatment.

**Results:**

After FDR correction, eight significant proteins were identified: IL7R, TYMP, IL12B, CCL8, TNFAIP6, IL18R1, IL23R, and ERAP1. Elevated levels of IL7R, IL12B, CCL8, IL18R1, IL23R, and ERAP1 increased AS risk, whereas elevated TYMP and TNFAIP6 levels decreased AS risk. Colocalization analysis indicated that IL23R, IL7R, and TYMP likely share causal variants with AS. PPI network analysis identified IL23R and IL7R as potential new therapeutic targets.

**Conclusions:**

This study identified eight plasma proteins with significant associations with AS risk, suggesting IL23R, IL7R, and TYMP as promising therapeutic targets. Further research is needed to explore underlying mechanisms and potential for drug repurposing.

## 1 Introduction

Ankylosing spondylitis (AS) is a disabling chronic arthritis resulting from a combination of factors. Genetic susceptibility does not fully explain the etiology of AS, and the effect of the interplay of genes, sex, microorganisms, mechanical stress, and additional lifestyle and environmental factors on increased susceptibility to AS is unclear, which adds to the complexity of treatment (1). Targeted biologic therapies are the mainstay treatment for patients with AS who are difficult to treat with nonsteroidal anti-inflammatory drugs (2). In recent years, the successful introduction of monoclonal antibodies against AS (interleukin [IL]-17A and tumor necrosis factor-α [TNF-α]) has exemplified numerous vital pathological pathways. Nonetheless, <50% of patients respond favorably to IL-17A and TNF-α blockade. Currently, curative treatment for AS is lacking, and many patients have to manage symptoms through lifelong medication, which can induce adverse reactions (3). Consequently, exploring novel diagnostic biomarkers and therapeutic agents is crucial to provide more effective diagnostic methods and therapeutic options for patients with AS, thereby improving patient prognosis.

Human proteins are crucial in diverse biological processes and represent the main drug targets. Nelson et al. (4)revealed that protein drug targets linked to genetic association-supported diseases are likely to be approved for marketing, with a possible twofold increase. In recent years, Mendelian randomization (MR) analyses have been extensively applied to develop and repurpose drug targets (5). Genetic instrumental variable analysis involves the use of single nucleotide polymorphisms (SNPs) from genome-wide association studies (GWAS) as the genetic approach to estimate the causal relationship between the exposure and the outcome. In contrast to observational studies, MR avoids the effects of confounding factors. Progress in plasma high-throughput genomic and proteomic technologies has allowed MR-based strategies to facilitate the identification of candidate therapeutic targets for diverse disorders (e.g., type 1 diabetes and breast cancer) (6, 7). However, MR analyses that integrate GWAS with protein quantitative trait locus (pQTL) data, an approach that could provide important insights for early disease diagnosis and drug target discovery, are lacking in AS research. Consequently, the present study aimed to integrate large-scale GWAS with pQTL data using MR analysis to investigate plasma proteins that could be candidate biomarkers or therapeutic targets of AS.

**Figure 1:**
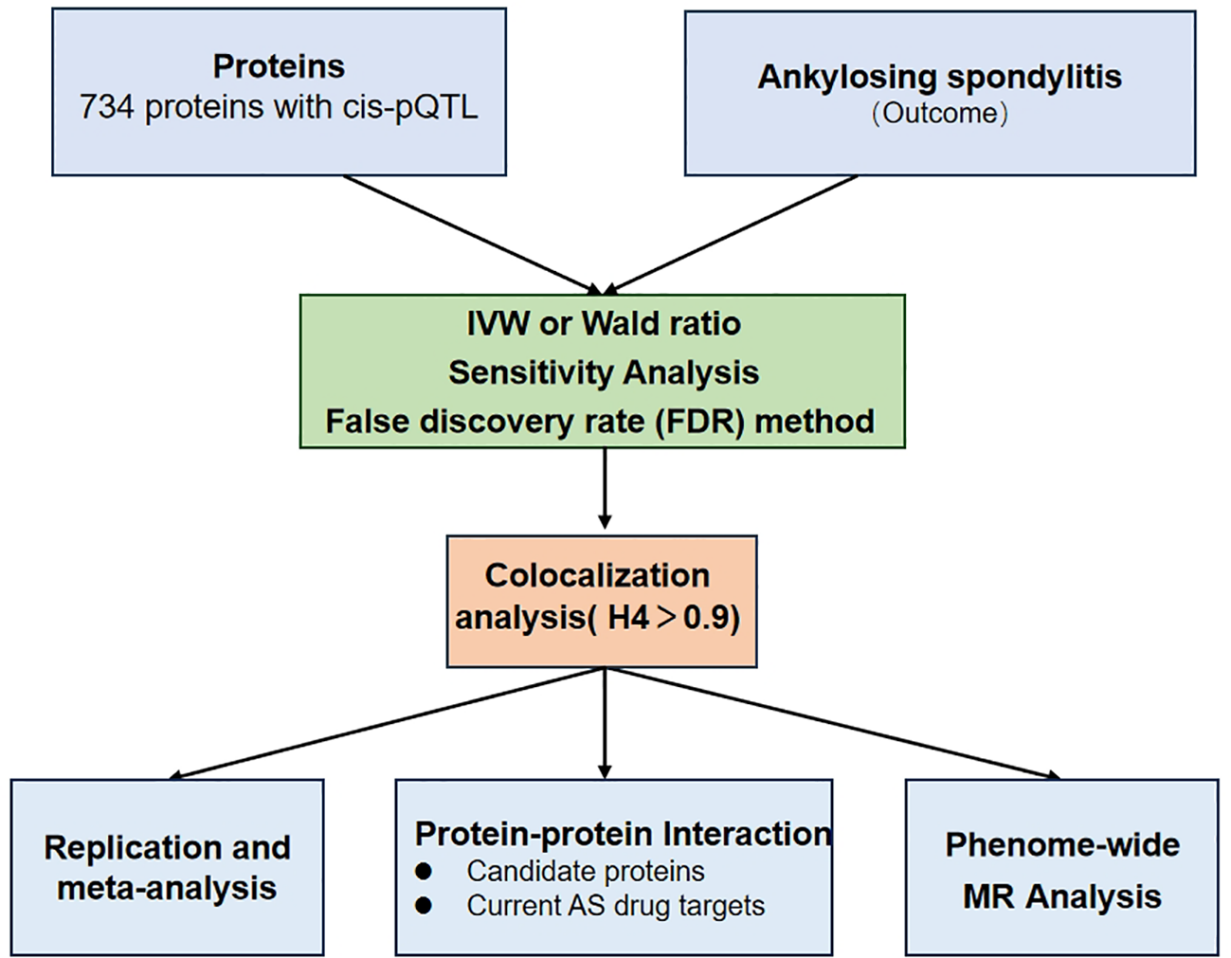
A flowchart for Mendelian randomization (MR) identification of pathogenic plasma proteins in ankylosing spondylitis (AS).

We established the precise control of AS by discovering potential drug targets from plasma proteins. **Figure 1** shows the study design. First, MR analysis of two samples was performed, and eight potential drug proteins were screened after FDR correction. The plasma protein data were extracted from plasma proteomics-related publications, whereas the AS data were obtained from the GWAS data of the International Genetics of Ankylosing Spondylitis Consortium (IGAS) (8). Secondly, we performed colocalization analysis to verify the robustness of the genetic associations between plasma proteins and AS. Third, the relationship between the identified proteins was analyzed using the protein-protein interaction (PPI) network to identify potential therapeutic targets. Fourth, a sensitivity analysis, including Replication and meta-analysis, was conducted to ensure the accuracy and directionality of the identified associations. Finally, we assessed the potential adverse effects of identified drug proteins on other phenotypes using phenome-wide Mendelian randomization analysis.

## 2 Methods

### 2.1 Exposure data acquisition

First, plasma protein data were obtained from recent publications in Nature Genetics (9), which integrated data from five previous GWAS datasets (10–14), to analyze 738 cis-pQTLs for 734 plasma proteins obtained using the following criteria (**Supplementary Table S1**):

i) Located outside of the major histocompatibility complex region (chr6, 26–34 Mb).
ii) Exhibiting noteworthy genome-wide associations (*p*-value < 5*10−8).
iii) Functioning as a cis-acting pQTL.
iv) Displaying autonomous association with linkage disequilibrium (LD) clumping (r2 <0.001).

Additionally, the F-statistic for each instrumental variable was determined using the formula: F = R2×(N − 2)/(1 − R2), where R2 is calculated as 2×EAF×(1 − EAF)×β2. This calculation helps to prevent biases due to weak instruments, and an F-statistic above 10 is deemed adequate to overcome such biases (15). SNPs with palindromic structures were systematically removed from the analysis.

### 2.2 Sources of outcome data

We obtained a GWAS summary dataset from the IGAS for the preliminary analysis of AS, which included 19,688 patients and 15,145 controls. GWAS summary data for Ankylosing spondylitis was also obtained from multiple independent cohort studies, all of European ancestry. No overlapping participating studies were shared between the GWASs for plasma protein levels and Ankylosing spondylitis.

### 2.3 MR analysis

Our analysis complies with the STROBE-MR guidelines, and we have included the comparative report as **Supplementary Table S6**. We performed Mendelian randomization studies to assess the causal links between plasma protein levels and Ankylosing spondylitis. In MR, genetic variants are used as proxies for risk factors. Therefore, the instrumental variables (IVs) chosen must meet three essential criteria to ensure valid causal inference: (1) the genetic variants must be directly linked to the exposure; (2) the genetic variants should not be linked to any confounders that could affect the relationship between the exposure and the outcome; (3) the genetic variants should affect the outcome only through the exposure and not via any other pathways.

Plasma proteins and AS were utilized as the exposure and outcome, respectively. MR analysis was conducted using “TwoSampleMR” (https://github.com/MRCIEU/TwoSampleMR). If there was only one pQTL for a given protein, the Wald ratio was used. If at least two genetic tools could be applied, inverse variance-weighted MR (IVW-MR) was used and subsequently analyzed for heterogeneity (16). In the preliminary analysis, we applied the false discovery rate (FDR) correction for multiple testing to select potentially effective causal proteins. A sensitivity analysis was performed to verify the accuracy of the data. For proteins to which at least two genetic tools could be applied, we performed heterogeneity analysis. Steiger filtering was also performed to verify the association direction of AS with the plasma proteins.

### 2.4 Colocalization analysis

Colocalization is an additional analysis that strengthens the results of genetic studies by seeking evidence of the same genetic variants being associated with both the exposure and the outcome. This helps to confirm that the results are due to a causal relationship with the genetic variant, rather than due to linkage disequilibrium (LD) or other confounding factors. We conducted colocalization analysis using the coloc R package (17). Colocalization analysis provides five hypotheses: H0, the genetic variant is not associated with either trait; H1, associated only with one trait; H2, associated only with the other trait; H3, associated with both traits but with different causal variants; and H4, associated with both traits and with the same causal variant. We focused on proteins with a combined posterior probability of association PPH4 of 0.90 or greater (18).

### 2.5 Protein-protein and protein-drug association analysis

We utilized a protein–protein interaction (PPI) network to assess relevant plasma protein targets that were significantly related to AS susceptibility. We constructed a functional protein interaction network (https://cn.string-db.org) from the STRING database. Typically, the minimal interaction score required for STRING was 0.4. Identifying protein targeting pathways is important for the discovery of efficient drug compounds that can change target or downstream protein activity to terminate disease development. Consequently, the dgidb database (https://dgidb.org/) was utilized to explore the relationships of AS protein targets with corresponding genes.

### 2.6 Phenome-wide MR

We used summary statistics of diseases from the UK Biobank cohort to perform phenome-wide Mendelian randomization analysis to investigate the potential side effects of these five candidate drug genes. To ensure the accuracy and scalability of the analysis, the UK Biobank disease GWAS employed the generalized mixed model (SAIGE V.0.29) method to address the issue of unbalanced case-control ratios (19). Based on statistical power considerations, we selected 783 traits (diseases) with at least 500 cases for phenotypic MR analysis. Subsequently, we conducted MR analysis using the IVW or Wald ratio method with the same parameters. If the FDR-corrected p-value was less than 0.05, the causal effect was considered statistically significant. The summary statistics for disease-associated SNPs were obtained from SAIGE GWAS (available for download at https://www.leelabsg.org/resources) (19).

### 2.7 Replication and meta-analysis

We repeated the MR analysis in another AS cohort, which included 1,462 cases of European ancestry and 164,682 controls of European ancestry, identifying 16,380,022 SNPs to comprehensively evaluate the robustness of the candidate proteins identified by the above criteria. The GWAS data used for replication analysis of AS was obtained from the Finnish database and included in the IEU GWAS repository (https://gwas.mrcieu.ac.uk/datasets/finn-b-M13_ANKYLOSPON/) with the GWAS ID finn-b-M13_ANKYLOSPON. The criteria for replication analysis were that the AS SNP had the same direction of effect and reached p<0.05 in the meta-analysis of the combined results of the two replication GWAS. The meta-analysis was conducted using the R package “meta” (version 7.0-0).

## 3 Results

### 3.1 Identification of significant proteins associated with ankylosing spondylitis

**Figure 2:**
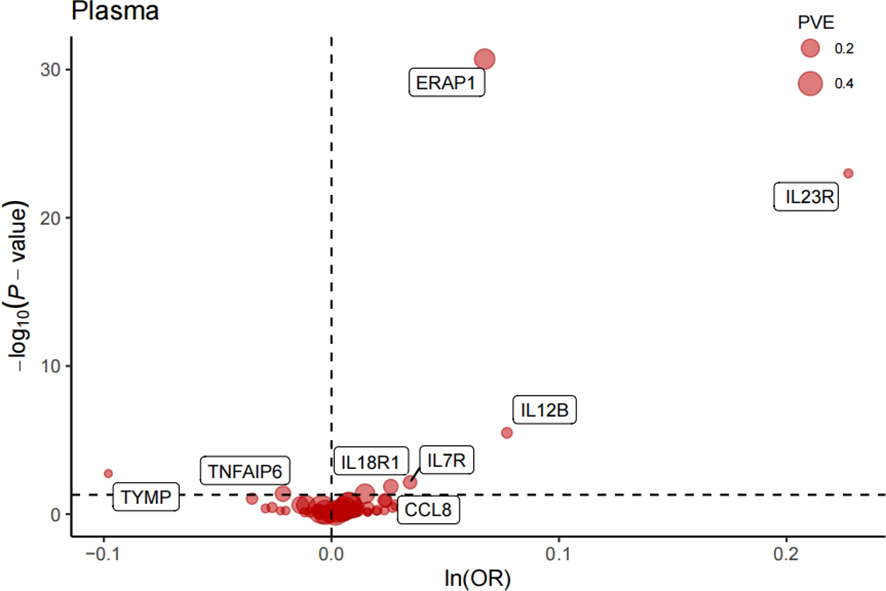
Volcano plot of MR analysis of 734 plasma proteins for AS risk. OR, odds ratio, per standard deviation increase in plasma protein levels. Dashed horizontal line represented P-fdr =0.05;PVE, proportion of variance explained.

After FDR correction, we identified eight significant proteins (**Table 1**, **Figure 2**), including interleukin 7 receptor (IL7R), thymidine phosphorylase (TYMP), interleukin 12B (IL12B), C-C motif chemokine ligand 8 (CCL8), TNF alpha-induced protein 6 (TNFAIP6), interleukin 18 receptor 1 (IL18R1), interleukin 23 receptor (IL23R), and endoplasmic reticulum aminopeptidase 1 (ERAP1).

**Table 1:** Mendelian randomization analysis of plasma protein and ankylosing spondylitis after FDR correction.

Protein	SNP	Method	OR (95% CI)	P value	PVE	F	Steiger_pval	PPH4
IL7R	rs11957503	Wald ratio	1.04 (1.01, 1.06)	7.12e-03	8.69%	94.74	3.474e-16	0.976
TYMP	rs131798	Wald ratio	0.91 (0.86, 0.95)	1.83e-03	1.42%	46.24	4.193e-05	0.932
IL12B	rs4921484	Wald ratio	1.08 (1.05, 1.11)	3.28e-06	4.13%	142.08	6.844e-15	0.108
CCL8	rs3138036	IVW	1.03 (1.01, 1.04)	1.39e-02	11.57%	61.20	1.646e-11	0.197
TNFAIP6	rs289828	Wald ratio	0.98 (0.96, 0.99)	4.21e-02	13.93%	534.45	4.725e-75	0.435
IL18R1	rs1420106	Wald ratio	1.01 (1.00, 1.03)	4.21e-02	27.46%	1249.64	1.648e-170	0.036
IL23R	rs11581607	Wald ratio	1.26 (1.20, 1.31)	1.01e-23	2.17%	73.17	0.016	0.998
ERAP1	rs17482078	Wald ratio	1.07 (1.06, 1.08)	1.94e-31	30.77%	1467.41	7.682e-144	<0.001

OR, odds ratio; per standard deviation increase in plasma protein levels. PVE, proportion of variance explained; IVW, inverse variance weighted.

Specifically, elevated IL7R (OR = 1.04, 95% CI: 1.01–1.06, P = 7.12e−03), IL12B (OR = 1.08, 95% CI: 1.05–1.11, P = 3.28e−06), CCL8 (OR = 1.03, 95% CI: 1.01–1.04, P = 1.39e−02), IL18R1 (OR = 1.01, 95% CI: 1.00–1.03, P = 4.21e−02), IL23R (OR = 1.26, 95% CI: 1.20–1.31, P = 1.01e−23), and ERAP1 (OR = 1.07, 95% CI: 1.06–1.08, P = 1.94e−31) increased AS risk. However, elevated TYMP (OR = 0.91, 95% CI: 0.86–0.95, P = 1.83e−03) and TNFAIP6 (OR = 0.98, 95% CI: 0.96–0.99, P = 4.21e−02) decreased AS risk. In addition, no heterogeneity was detected in the analyzed plasma proteins, such as the heterogeneity test for CCL 8 (pval = 0.979).The detailed main MR analysis results are shown in **Supplementary Table S2**. The Steiger filtering method utilizes a statistical test to pinpoint the stronger bidirectional effects. The findings indicated that the eight proteins identified in the primary analysis did not corroborate the presence of reverse causal effects (**Table 1**).

### 3.2 Colocalization analysis of the eight significant proteins

**Figure 3:**
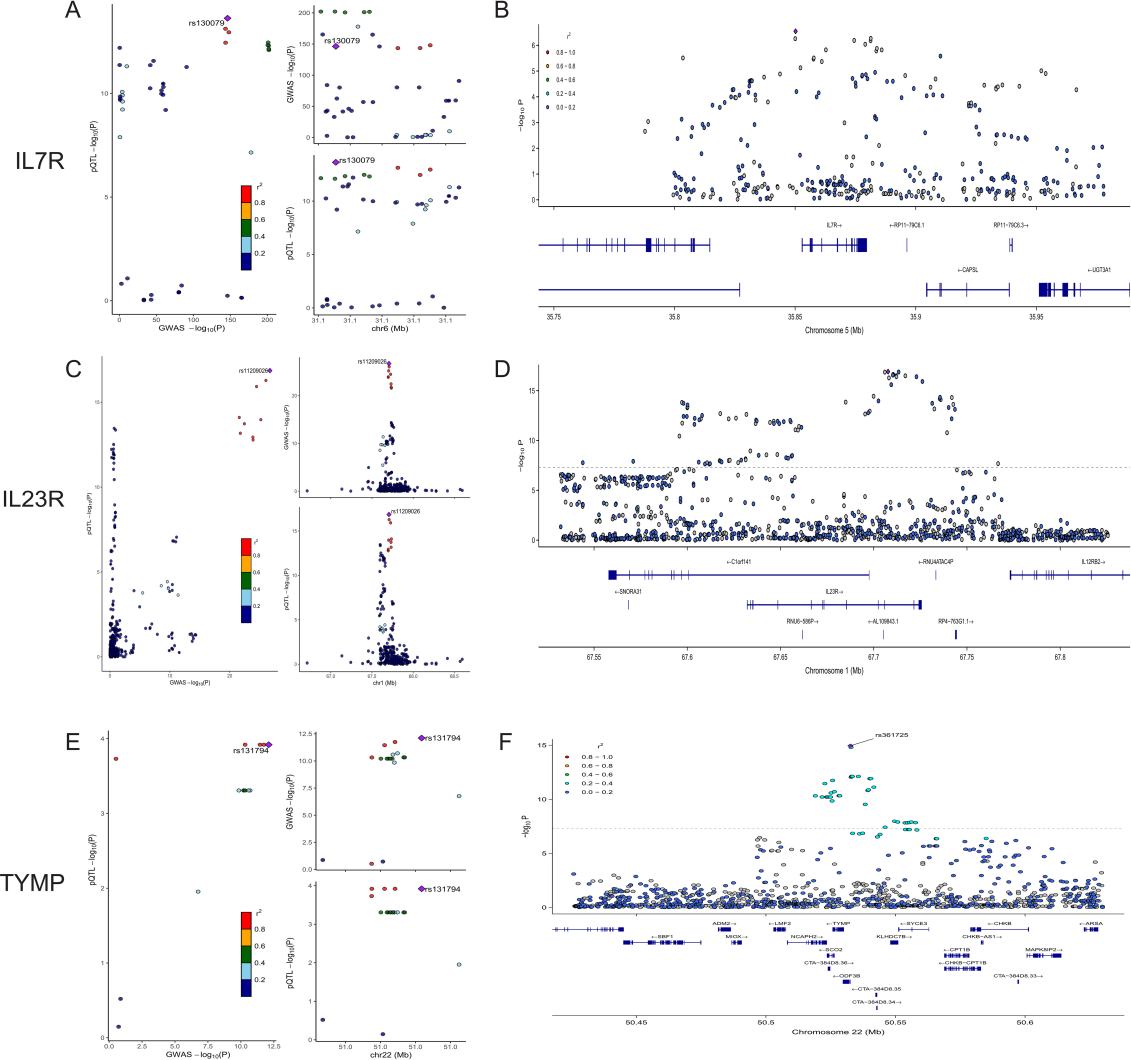
Colocalization and Gene Track Plots for IL7R, IL23R, and TYMP. **(A, C, E)** display the colocalization plots for IL7R, IL23R, and TYMP. **(B, D, F)** present the corresponding gene track plots for these proteins, illustrating the -log10(P-value) along the chromosomal position. The gene locations and structures are shown below the association signals.

We conducted colocalization analyses for the eight candidate proteins to further determine the likelihood of shared causal genetic variants associated with AS and pQTL. The results indicate that IL23R, IL7R, and TYMP are likely to share causal variants in this region (PPH4 > 0.90), making them the strongest candidate proteins for AS (**Figure 3**, **Supplementary Table S3**). On the other hand, ERAP1, IL18R1, CCL8, TNFAIP6, and IL12B are less likely to share causal variants with AS in this region (PPH4 < 0.90). The colocalization and genes track plots for these five proteins are shown in **Supplementary Figure S1**. Notably, although the PPH4 values for ERAP1 and IL18R1 are less than 0.9, their PPH3 values are close to 1. Thus, we believe that these two genes are associated with protein levels in this region and AS, but the evidence supports separate causal variants.

### 3.3 Relationships between candidate drug targets and AS

The PPI networks illustrate the interactions of three prioritized proteins (IL23R, IL7R,and TYMP) with two current AS drug targets (TNF-α and IL17), as shown in **Supplementary Figure S2**. Specifically, IL23R and IL7R are associated with TNF-α, which is targeted by infliximab and adalimumab. Additionally, the PPI network shows that IL23R and IL7R are associated with IL17, a target of ixekizumab (**Supplementary Figure S3**). This indicates the potential relevance of IL23R and IL7R as new therapeutic targets for AS, supported by their interactions with existing drug targets. We searched the DGIdb database (https://dgidb.org/) for current drugs targeting potential disease-causing proteins. Three potential disease-modifying drugs were identified: tipiracil hydrochloride targeting Thymidine Phosphorylase (TYMP), ruxolitinib targeting Interleukin 7 Receptor (IL7R), and celecoxib targeting Interleukin 23 Receptor (IL23R). All these medications are approved, highlighting their potential for repurposing in AS treatment (**Supplementary Table S5**).

### 3.4 Phenome-wide MR analysis of candidate drug-target proteins

**Figure 4:**
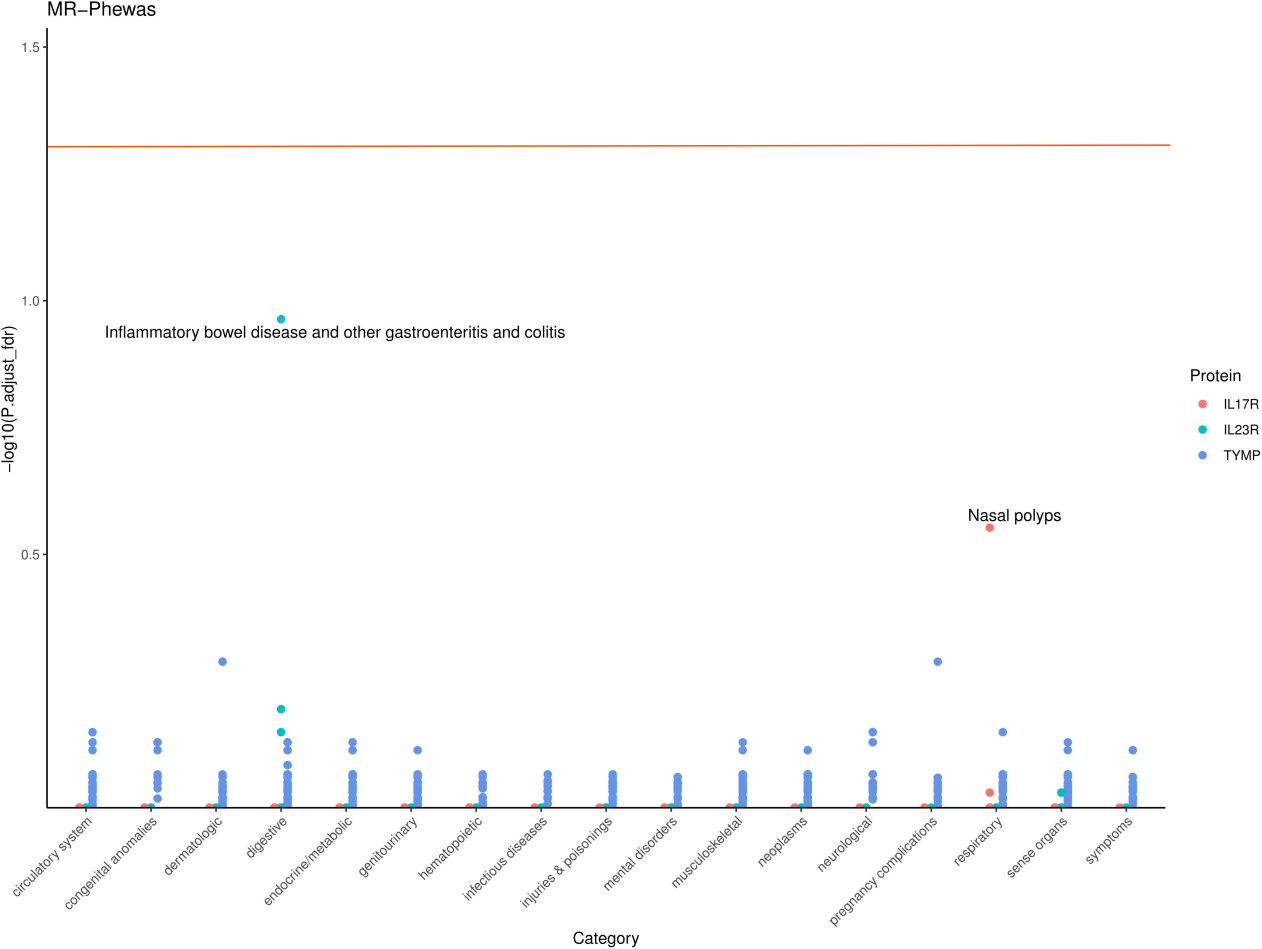
Phenome-wide Association Study (PheWAS) of IL17R, IL23R, and TYMP. This scatter plot displays the -log10 (P,adjust_fdr) for the associations between IL17R, IL23R, and TYMP gene levels and various phenotypes across different categories. Each point represents a specific phenotype within a category, with colors indicating the corresponding protein. The horizontal line represents the significance threshold.

To assess the potential beneficial or harmful effects of these three AS-related candidate proteins on other phenotypes, we conducted a phenome-wide association study. A comprehensive MR screening of 783 diseases or traits was performed using data from the UK Biobank. Overall, we identified 86 phenotypes that may have a causal relationship with the candidate proteins (P < 0.05), as shown in **Supplementary Table S4**. After FDR correction, there was almost no statistical evidence of adverse side effects for these candidate drug proteins, suggesting that their development appears to be safe (**Figure 4**).

### 3.5 Replication and meta-analysis

**Figure 5:**
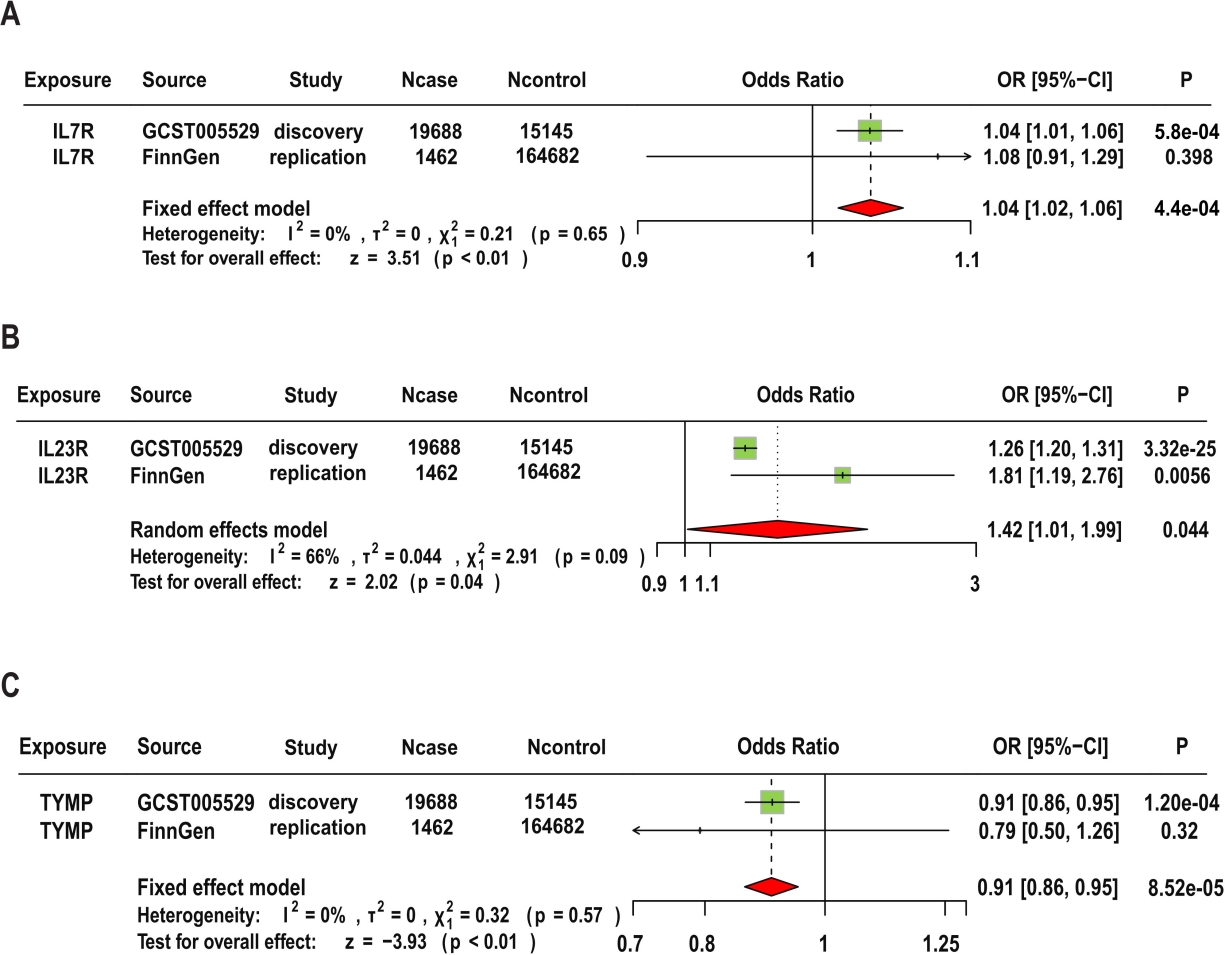
Replication and Meta-Analysis of Three Candidate Proteins. Meta-analysis of IL7R **(A)**, IL23R **(B)**, and TYMP **(C)** using data from FinnGen cohorts.

In the replication analysis, we used the original protein SNPs (**Supplementary Table S1**) as exposure instruments and AS data from the Finnish database as the outcome for MR analysis. The main analysis methods remain the Wald ratio or IVW. Additionally, we conducted a meta-analysis combining results from the replication cohort and the original results. Our criteria for replication analysis are: the direction of effect of the targeted protein on disease risk must be consistent (i.e., the sign of beta in both MR analyses should be the same), and the meta-analysis of the two replication GWAS results should reach p<0.05. In the replication cohort, we found that the MR results for IL23R were still significant (OR=1.81, 95% CI (1.19, 2.76), P=0.005), but it should be noted that IL7R and TYMP did not show statistically significant evidence in the replication cohort. The meta-analysis results indicate that although the combined p-values for IL7R and IL23R were significantly attenuated when discovery and replication results were combined, the MR results for the three candidate proteins remained significant. Specifically, IL23R (OR=1.42, 95% CI: 1.01–1.99, P-meta=0.044), IL7R (OR=1.04, 95% CI: 1.02–1.06, P-meta=0.00044), and TYMP (OR=0.91, 95% CI: 0.86–0.95, P-meta=8.52e-5). Additionally, the replication results for these three target proteins were consistent with the findings regarding disease risk. The detailed results are shown in **Figure 5**.

### 3.6 Comparison with the study by Zhao et al.

**Figure 6:**
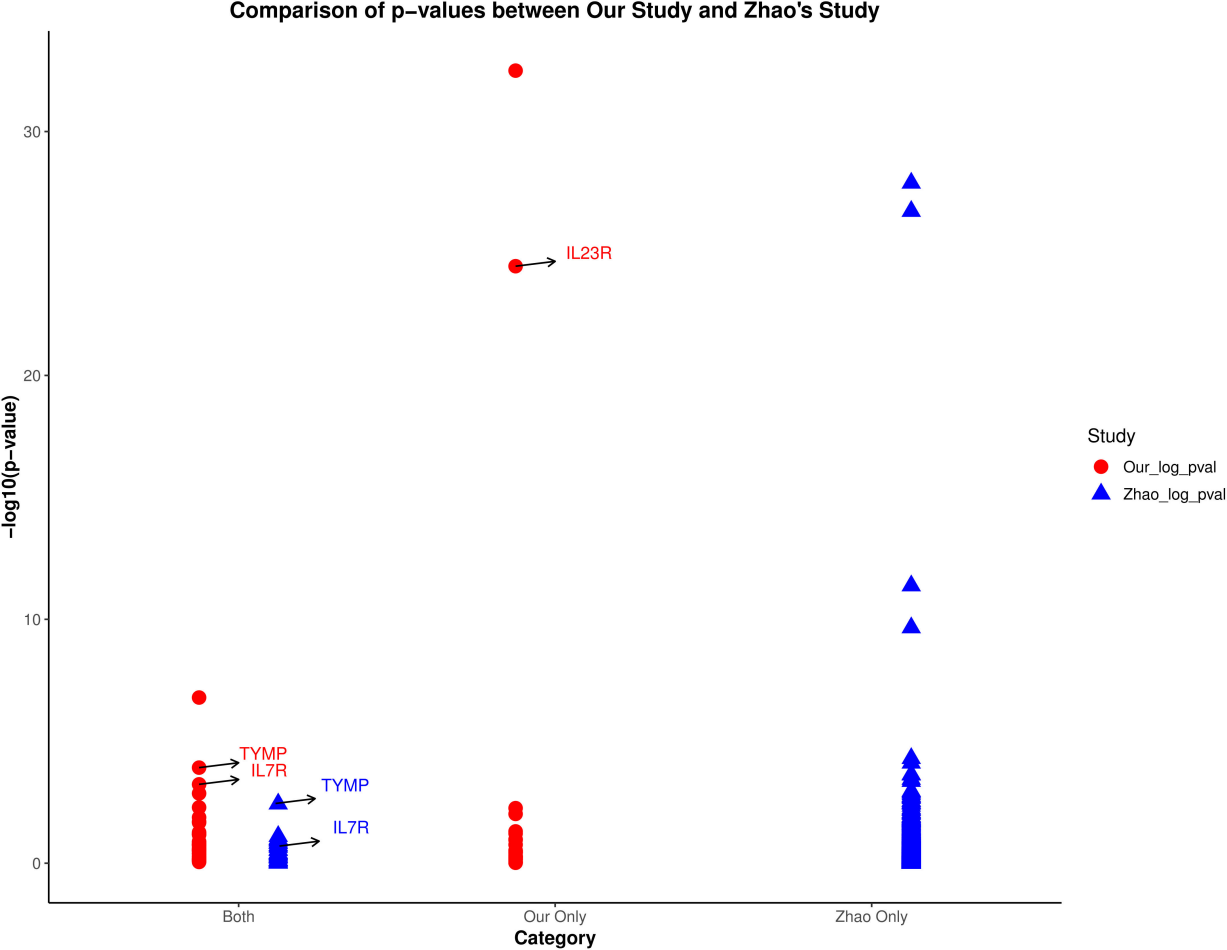
Comparison of p-values between our study and Zhao et al.’s study. The plot categorizes proteins into three groups: identified in both studies (Both), only in our study (Our Only), and only in Zhao et al.’s study (Zhao Only). Highlighted are the p-values of candidate proteins IL23R, TYMP, and IL7R.

The proteomics data used by Zhao et al. (20)came from the UK Biobank-PPP database, and the AS genetic association study data came from the R9 version of the Finnish database. Their study did not include a replication cohort. Given that the protein pQTL data and outcomes used in Zhao et al.’s study are different from those used in our study, we conducted a comparative analysis of their results. **Figure 6** illustrates a comparison of protein MR effect estimates between our study and Zhao et al.’s study, categorizing proteins into three groups: proteins identified in both studies (Both), proteins identified only in our study (Our Only), and proteins identified only in Zhao et al.’s study (Zhao Only). We highlighted the p-values of the candidate proteins (IL23R, TYMP, and IL7R) in both studies. The figure clearly shows that TYMP and IL7R have more significant p-values in our study compared to Zhao et al.’s study. In their study, the MR effect of TYMP was available but not significant in the UK Biobank, which explains the absence of these two proteins in Zhao et al.’s results. As for IL23R, its absence in Zhao et al.’s study is due to the lack of measurement of this protein in the UK Biobank. Therefore, our research serves as an extension and enhancement of Zhao et al.’s findings.

## 4 Discussion

The development of new therapeutic agents for ankylosing spondylitis is challenging. One of the main reasons for this difficulty is the incomplete understanding of the pathophysiology of AS. The human proteome is the primary therapeutic target, and protein drug targets possess significant clinical value. Therefore, an integrative analysis was conducted to identify novel anti-AS therapeutic targets to evaluate the causative proteins of AS based on prior GWAS (21).

The “causality” detected through MR could be genetic confounding, or horizontal pleiotropy because of LD (9). Thus, only cis-pQTL were used as instruments to limit horizontal pleiotropy bias because they directly affect the translation and transcription of the gene of interest. In this study, we analyzed the potential pathogenic relationship between plasma proteins and the risk of AS and identified potential therapeutic targets among plasma proteins for AS. After adjusting for FDR, the plasma proteins ERAP1, IL12B, IL18R1, IL23R, IL7R, CCL8, TNFAIP6, and TYMP showed a causal relationship with the risk of AS. The Steiger filtering method were used to validate the directionality of the causal relationships (22). These methods showed no evidence of reverse causation for the proteins identified in the primary analysis. This reinforces the finding that IL23R, IL7R, and TYMP are likely contributors to the pathogenesis of AS, suggesting that these proteins play a causative role in the disease’s development rather than being a consequence of it. The PPI networks revealed that IL23R and IL7R interact with current AS drug targets TNF-α and IL17, indicating their potential as new therapeutic targets. Additionally, database searches identified three approved drugs—tipiracil hydrochloride, ruxolitinib, and celecoxib—targeting TYMP, IL7R, and IL23R, respectively, suggesting their potential for repurposing in AS treatment (23). In the replication analysis section, we attempted to replicate the study results using FinnGen AS GWAS as the outcome. We found that the MR result for IL23R remained significant in the replication analysis. As for IL23R and TYMP, although the direction of the MR results was consistent with the discovery results, they did not reach significance in the replication cohort. This may be due to the smaller sample size of the replication cohort, which reduces statistical power. In addition, our MR PheWAS results indicate that developing these potential drug proteins appears to be safe, as there is little statistical evidence to suggest that they will produce harmful side effects (24).

IL23R is implicated in the IL-23/IL-17 axis, which plays a crucial role in the pathogenesis of AS. Studies have shown that IL-23 stimulates the expansion and maintenance of Th17 cells, leading to increased levels of IL-17, a proinflammatory cytokine involved in AS (25, 26). Genetic variants in IL23R have been associated with AS susceptibility, highlighting it as a potential therapeutic target. IL7R is essential for T-cell development and homeostasis. Variants in IL7R have been linked to multiple autoimmune diseases, including AS. The IL-7 receptor is involved in the survival and proliferation of T cells, which are critical in the inflammatory processes underlying AS (27). Targeting IL7R could modulate immune responses and potentially ameliorate AS symptoms. Of the eight AS-related proteins identified in the present study, ERAP1, IL12B, and IL23R were shown to be related to AS via numerous animal experiments and clinical studies, with ERAP1 suggested to be the second most potent gene related to AS (28, 29). This further establishes the reliability of our study of ERAP 1 as a drug target for treatment of AS. ERAP1 and HLA-B27 contribute to 70% of familial genetic factors for AS (30). ERAP1 polymorphisms have been shown to affect AS susceptibility in HLA-B27+ people (31). Thus, ERAP1 can exert synergistic effects on factors such as HLA-B27 and is related to abnormal peptide processing and incorrect antigen presentation, leading to AS susceptibility. According to one case−control association study, protective genetic variants are related to a decrease in ERAP1 and ERAP2 function and inhibition of cell surface major histocompatibility complex I expression (8). ERAP1 and ERAP2 variants can affect associated peptide numbers to decrease the accelerated HLA-B27 folding rate, thus aggravating ER stress while accelerating AS progression (32). Mei et al. (33) reported that AS patients had higher serum IL-17 and IL-23 levels than did normal participants. IL-23, an IL-12-associated cytokine, may promote T helper (Th)17 cell growth and differentiation (34). Th17 cells are involved in the pathogenesis of AS (35) and are also implicated in psoriasis, inflammatory arthritis, and Crohn’s disease (36–38). IL-12B is likely related to the pathogenesis of AS because of its effects on IL-23R+CD4+ T cells and preferential stimulation of the above cells to release IL-17 predominantly (39). The present systematic study based on MR studies precisely addresses the inability of previous animal experiments to infer causality. Moreover, we identified novel AS-associated proteins (TYMP, CCL8, and TNFAIP6). TNFAIP6, also known as TSG 6, is a class of proteins induced by TNF-α that is secreted during acute inflammatory responses and is associated with inflammation and tissue remodeling (40). Previous studies have reported that TNFAIP6 has anti-inflammatory effects in an experimental mouse model of arthritis (41), but further evidence to support this observation is lacking. Moreover, the present study suggested that TNFAIP6 may have similar anti-inflammatory protective effects on AS via MR, providing a strong reference for the TNFAIP family as the causative genes for AS. CCL8 is a monocyte chemokine that can interact with CCR1, CCR2B, and CCR3; it also regulates tumor occurrence, antiviral infections, and inflammatory immunity in the host (42). Consistent with the present findings, one study suggested that CCL8 is upregulated in the serum of patients with AS and may be a useful biomarker for predicting active inflammation in the sacroiliac joints of patients with AS (43). TYMP catalyzes reversible thymidine phosphorylation and is suggested to have a critical effect on angiogenesis, tumor growth, migration, and invasion (44). A recent study showed that TNF-α strongly stimulates TYMP expression in fibroblast-like synoviocytes. Thus, we hypothesize that TNF-α may affect AS by inducing TYMP expression.

Previously, Zhao et al. (20)studied drug targets for ankylosing spondylitis using the UK Biobank-PPP database. Given that the pQTL data used by Zhao et al. differs from the data source in our study, we conducted a comparative analysis of their findings. The results indicate that the target proteins we identified are entirely different from theirs. For instance, the absence of IL23R protein measurement in the UK Biobank led to its omission in Zhao et al.’s study, thereby extending the data coverage scope in our research. Additionally, the MR analysis results for TYMP in Zhao et al.’s study indicated potential significance (P=0.0037), suggesting a potential causal role of this protein in the risk of AS. However, after p-value correction, TYMP was not selected as a candidate drug protein in their study. Furthermore, our discovery of drug-target candidate proteins (IL23R, TYMP, and IL7R) passed rigorous screening, with co-localization analysis strongly supporting their shared causal variants with AS in the region (PPH4 > 0.90). Moreover, our MR-PheWAS results showed almost no statistical evidence of adverse side effects for these candidate drug proteins, indicating that their development seems to be safe. Overall, these findings demonstrate the feasibility and practicality of our candidate proteins in drug development. We believe that the screening of these targeted proteins and the analysis of adverse effects can provide valuable references for research teams developing new targeted drugs for AS.

However, this study has certain limitations. First, since the instrumental variables included in this study mainly consist of a single cis-acting SNP and lack trans-pQTLs, it is not possible to determine the sensitivity test results at this level, which affects pleiotropy and heterogeneity analyses. Nevertheless, the F-statistics for our selected SNPs were all greater than 10, indicating minimal weak instrument bias. Second, the coloc method assumes single causal variants, which may not be accurate, and we are unable to assess AS progression genes (as GWAS identifies AS susceptibility genes). Therefore, our focus is on finding targets for disease prevention rather than treatment. Third, this study’s exposure and outcome data came from groups with European heritage. To translate these findings into practical applications, more study on non-European ancestries is needed in order to apply the conclusions to other locations, including Asia, Africa, and the Americas. Fourth, the results of the PPI study are suggestive rather than definitive, despite the fact that we were able to identify certain connections between pathogenic proteins and therapeutic targets of existing MS medications. It is need to do further research involving people who are healthy and patients with AS to confirm these correlations.

## 5 Conclusions

Our study identified eight plasma proteins significantly associated with ankylosing spondylitis risk using Mendelian randomization and colocalization analyses. We provided robust evidence for the causal roles of IL23R, IL7R, and TYMP, highlighting them as promising therapeutic targets. Phenome-wide MR analysis also evaluated potential side effects of these targets. Despite limitations, our findings enhance understanding of the genetic architecture of AS and support future research to validate these targets for clinical application.

## Data Availability

The datasets presented in this study can be found in online repositories. The names of the repository/repositories and accession number(s) can be found in the article/**Supplementary Material**.

## Supplementary material

Supplementary Figure S1 Colocalization and Gene Track Plots for Five Proteins. **(A, C, E, G, I)** display the colocalization plots for IL12B, ERAP1, IL18R1, CCL8, and TNFAIP6, respectively, showing the -log10(P-value) from the GWAS (x-axis) against the -log10(P-value) from the pQTL analysis (y-axis), with colors representing the linkage disequilibrium (LD) r² values. **(B, D, F, H, J)** present the corresponding gene track plots for these proteins, illustrating the -log10(P-value) along the chromosomal position, indicating genetic variants associated with AS and the candidate proteins. The gene locations and structures are shown below the association signals.

Supplementary Figure S2 Protein-protein interaction networks showing the interactions between three prioritized proteins (IL23R, IL7R, and TYMP) and two current AS drug targets (TNF and IL17).

Supplementary Figure S3 Protein-Protein Interaction (PPI) Networks of AS Medications, Drug Targets, and Potential Targets. This diagram illustrates the interactions between current ankylosing spondylitis (AS) medications, their drug targets, and potential new therapeutic targets. AS medications (infliximab, adalimumab, golimumab, and ixekizumab) are shown to target TNF-α and IL17. The potential targets identified are IL23R and IL7R, which are connected to the existing drug targets through co-expression (red lines) and text mining evidence (green lines).

## References

[1] Hwang MC Ridley L Reveille JD. Ankylosing spondylitis risk factors: a systematic literature review. Clin Rheumatol. (2021) 40:3079–93. doi: 10.1007/s10067-021-05679-7 PMC9044547 33754220

[2] Kenyon M Maguire S Rueda Pujol A O'Shea F McManus R. The genetic backbone of ankylosing spondylitis: how knowledge of genetic susceptibility informs our understanding and management of disease. Rheumatol Int. (2022) 42:2085–95. doi: 10.1007/s00296-022-05174-5 PMC9548471 35939079

[3] Nancy Z Yan L Hui S Paul B Liye C. From the genetics of ankylosing spondylitis to new biology and drug target discovery. Front Immunol. (2021) 12:624632. doi: 10.3389/fimmu.2021.624632 33679768 PMC7925991

[4] Nelson MR Tipney H Painter JL Shen J Nicoletti P Shen Y. The support of human genetic evidence for approved drug indications. Nat Genet. (2015) 47:856–60. doi: 10.1038/ng.3314 26121088

[5] Reay WR Cairns MJ. Advancing the use of genome-wide association studies for drug repurposing. Nat Rev Genet. (2021) 22:658–71. doi: 10.1038/s41576-021-00387-z 34302145

[6] Yazdanpanah N Yazdanpanah M Wang Y Forgetta V Pollak M Polychronakos C. Clinically relevant circulating protein biomarkers for type 1 diabetes: Evidence from a two-sample mendelian randomization study. Diabetes Care. (2022) 45:169–77. doi: 10.2337/dc21-1049 34758976

[7] Zhang N Li Y Sundquist J Sundquist K Ji J. Identifying actionable druggable targets for breast cancer: Mendelian randomization and population-based analyses. EBioMedicine. (2023) 98:104859. doi: 10.1016/j.ebiom.2023.104859 38251461 PMC10628347

[8] Cortes A Hadler J Pointon JP Robinson PC Karaderi T Leo P. Identification of multiple risk variants for ankylosing spondylitis through high-density genotyping of immune-related loci. Nat Genet. (2013) 45:730–8. doi: 10.1038/ng.2667 PMC3757343 23749187

[9] Zheng J Haberland V Baird D Walker V Haycock PC Hurle MR. Phenome-wide Mendelian randomization mapping the influence of the plasma proteome on complex diseases. Nat Genet. (2020) 52:1122–31. doi: 10.1038/s41588-020-0682-6 PMC7610464 32895551

[10] Suhre K Arnold M Bhagwat AM Cotton RJ Engelke R Raffler J. Connecting genetic risk to disease end points through the human blood plasma proteome. Nat Commun. (2017) 8:14357. doi: 10.1038/ncomms14357 28240269 PMC5333359

[11] Sun BB Maranville JC Peters JE Stacey D Staley JR Blackshaw J. Genomic atlas of the human plasma proteome. Nature. (2018) 558:73–9. doi: 10.1038/s41586-018-0175-2 PMC6697541 29875488

[12] Yao C Chen G Song C Keefe J Mendelson M Huan T. Genome-wide mapping of plasma protein QTLs identifies putatively causal genes and pathways for cardiovascular disease. Nat Commun. (2018) 9:3268. doi: 10.1038/s41467-018-05512-x 30111768 PMC6093935

[13] Emilsson V Ilkov M Lamb JR Finkel N Gudmundsson EF Pitts R. Co-regulatory networks of human serum proteins link genetics to disease. Science. (2018) 361:769–73. doi: 10.1126/science.aaq1327 PMC6190714 30072576

[14] Folkersen L Fauman E Sabater-Lleal M Strawbridge RJ Frånberg M Sennblad B. Mapping of 79 loci for 83 plasma protein biomarkers in cardiovascular disease. PLoS Genet. (2017) 13:e1006706. doi: 10.1371/journal.pgen.1006706 28369058 PMC5393901

[15] Pierce BL Ahsan H Vanderweele TJ. Power and instrument strength requirements for Mendelian randomization studies using multiple genetic variants. Int J Epidemiol. (2011) 40:740–52. doi: 10.1093/ije/dyq151 PMC3147064 20813862

[16] Deng YT Ou YN Wu BS Yang YX Jiang Y Huang YY. Identifying causal genes for depression via integration of the proteome and transcriptome from brain and blood. Mol Psychiatry. (2022) 27:2849–57. doi: 10.1038/s41380-022-01507-9 35296807

[17] Giambartolomei C Vukcevic D SChadt EE Franke L Hingorani AD Wallace C. Bayesian test for colocalisation between pairs of genetic association studies using summary statistics. PLoS Genet. (2014) 10:e1004383. doi: 10.1371/journal.pgen.1004383 24830394 PMC4022491

[18] Su WM Gu XJ Dou M Duan QQ Jiang Z Yin KF. Systematic druggable genome-wide Mendelian randomisation identifies therapeutic targets for Alzheimer's disease. J Neurol Neurosurg Psychiatry. (2023) 94:954–61. doi: 10.1136/jnnp-2023-331142 PMC10579488 37349091

[19] Zhou W Nielsen JB Fritsche LG Dey R Gabrielsen ME Wolford BN. Efficiently controlling for case-control imbalance and sample relatedness in large-scale genetic association studies. Nat Genet. (2018) 50:1335–41. doi: 10.1038/s41588-018-0184-y PMC6119127 30104761

[20] Zhao W Fang P Lai C Xu X Wang Y Liu H. Proteome-wide Mendelian randomization identifies therapeutic targets for ankylosing spondylitis. Front Immunol. (2024) 15:1366736. doi: 10.3389/fimmu.2024.1366736 38566994 PMC10985162

[21] McGowan LM Davey Smith G Gaunt TR Richardson TG. Integrating Mendelian randomization and multiple-trait colocalization to uncover cell-specific inflammatory drivers of autoimmune and atopic disease. Hum Mol Genet. (2019) 28:3293–300. doi: 10.1093/hmg/ddz155 PMC6859431 31276585

[22] Xue H Pan W. Inferring causal direction between two traits in the presence of horizontal pleiotropy with GWAS summary data. PLoS Genet. (2020) 16:e1009105. doi: 10.1371/journal.pgen.1009105 33137120 PMC7660933

[23] Delen E Doganlar O Doganlar ZB Delen O. Inhibition of the Invasion of Human Glioblastoma U87 Cell Line by Ruxolitinib: A Molecular Player of miR-17 and miR-20a Regulating JAK/STAT Pathway. Turk Neurosurg. (2020) 30:182–9. doi: 10.1371/journal.pgen.1008785 31452174

[24] Bretherick AD Canela-Xandri O Joshi PK Clark DW Rawlik K Boutin TS. Linking protein to phenotype with Mendelian Randomization detects 38 proteins with causal roles in human diseases and traits. PLoS Genet. (2020) 16:e1008785. doi: 10.1371/journal.pgen.1008785 32628676 PMC7337286

[25] Cua DJ Sherlock J Chen Y Murphy CA Joyce B Seymour B. Interleukin-23 rather than interleukin-12 is the critical cytokine for autoimmune inflammation of the brain. Nature. (2003) 421:744–8. doi: 10.1038/nature01355 12610626

[26] Veldhoen M Uyttenhove C van Snick J Helmby H Westendorf A Buer J. Transforming growth factor-beta 'reprograms' the differentiation of T helper 2 cells and promotes an interleukin 9-producing subset. Nat Immunol. (2008) 9:1341–6. doi: 10.1038/ni.1659 18931678

[27] Wordsworth BP Cohen CJ Davidson C Vecellio M. Perspectives on the genetic associations of ankylosing spondylitis. Front Immunol. (2021) 12:603726. doi: 10.3389/fimmu.2021.603726 33746951 PMC7977288

[28] Zhu W He X Cheng K Zhang L Chen D Wang X. Ankylosing spondylitis: etiology, pathogenesis, and treatments. Bone Res. (2019) 7:22. doi: 10.1038/s41413-019-0057-8 31666997 PMC6804882

[29] Alvarez-Navarro C López de Castro JA. ERAP1 in ankylosing spondylitis: genetics, biology and pathogenetic role. Curr Opin Rheumatol. (2013) 25:419–25. doi: 10.1097/BOR.0b013e328362042f 23656713

[30] Tsui FW Haroon N Reveille JD Rahman P Chiu B Tsui HW. Association of an ERAP1 ERAP2 haplotype with familial ankylosing spondylitis. Ann Rheum Dis. (2010) 69:733–6. doi: 10.1136/ard.2008.103804 19433412

[31] Evans DM Spencer CC Pointon JJ Su Z Harvey D Kochan G. Interaction between ERAP1 and HLA-B27 in ankylosing spondylitis implicates peptide handling in the mechanism for HLA-B27 in disease susceptibility. Nat Genet. (2011) 43:761–7. doi: 10.1038/ng.873 PMC3640413 21743469

[32] Reveille JD. An update on the contribution of the MHC to AS susceptibility. Clin Rheumatol. (2014) 33:749–57. doi: 10.1007/s10067-014-2662-7 PMC4488903 24838411

[33] Mei Y Pan F Gao J Ge R Duan Z Zeng Z. Increased serum IL-17 and IL-23 in the patient with ankylosing spondylitis. Clin Rheumatol. (2011) 30:269–73. doi: 10.1007/s10067-010-1647-4 21161669

[34] Nady S Ignatz-Hoover J Shata MT. Interleukin-12 is the optimum cytokine to expand human Th17 cells in vitro. Clin Vaccine Immunol. (2009) 16:798–805. doi: 10.1128/CVI.00022-09 19386801 PMC2691060

[35] Shen H Goodall JC Hill Gaston JS. Frequency and phenotype of peripheral blood Th17 cells in ankylosing spondylitis and rheumatoid arthritis. Arthritis Rheum. (2009) 60:1647–56. doi: 10.1002/art.24568 19479869

[36] Fitch E Harper E Skorcheva I Kurtz SE Blauvelt A. Pathophysiology of psoriasis: recent advances on IL-23 and Th17 cytokines. Curr Rheumatol Rep. (2007) 9:461–7. doi: 10.1007/s11926-007-0075-1 PMC2893221 18177599

[37] Schmechel S Konrad A Diegelmann J Glas J Wetzke M Paschos E. Linking genetic susceptibility to Crohn's disease with Th17 cell function: IL-22 serum levels are increased in Crohn's disease and correlate with disease activity and IL23R genotype status. Inflammation Bowel Dis. (2008) 14:204–12. doi: 10.1002/ibd.20315 18022867

[38] Miossec P. Interleukin-17 in fashion, at last: ten years after its description, its cellular source has been identified. Arthritis Rheum. (2007) 56:2111–5. doi: 10.1002/art.22733 17599728

[39] Kikly K Liu L Na S Sedgwick JD. The IL-23/Th(17) axis: therapeutic targets for autoimmune inflammation. Curr Opin Immunol. (2006) 18:670–5. doi: 10.1016/j.coi.2006.09.008 17010592

[40] Wisniewski HG Naime D Hua JC Vilcek J Cronstein BN. TSG-6, a glycoprotein associated with arthritis, and its ligand hyaluronan exert opposite effects in a murine model of inflammation. Pflugers Arch. (1996) 431:R225–6. doi: 10.1007/BF02346350 8739346

[41] Bárdos T Kamath RV Mikecz K Glant TT. Anti-inflammatory and chondroprotective effect of TSG-6 (tumor necrosis factor-alpha-stimulated gene-6) in murine models of experimental arthritis. Am J Pathol. (2001) 159:1711–21. doi: 10.1016/s0002-9440(10)63018-0 PMC1867074 11696432

[42] Zhang X Chen L Dang WQ Cao MF Xiao JF Lv SQ. CCL8 secreted by tumor-associated macrophages promotes invasion and stemness of glioblastoma cells via ERK1/2 signaling. Lab Invest. (2020) 100:619–29. doi: 10.1038/s41374-019-0345-3 31748682

[43] Li X Liang A Cui Y Liao J Fang X Zhong S. Role of macrophage-associated chemokines in the assessment of initial axial spondyloarthritis. Clin Rheumatol. (2022) 41:3383–9. doi: 10.1007/s10067-022-06308-7 35882716

[44] Li W Yue H. Thymidine phosphorylase: A potential new target for treating cardiovascular disease. Trends Cardiovasc Med. (2018) 28:157–71. doi: 10.1016/j.tcm.2017.10.003 PMC5856583 29108898

