# Supplementary figures and images for: Investigating potential novel therapeutic targets and biomarkers for ankylosing spondylitis using plasma protein screening
Source: Front Immunol. 2024 Aug 9;15:1406041. doi: 10.3389/fimmu.2024.1406041 (PMC11341372; doi:10.3389/fimmu.2024.1406041)

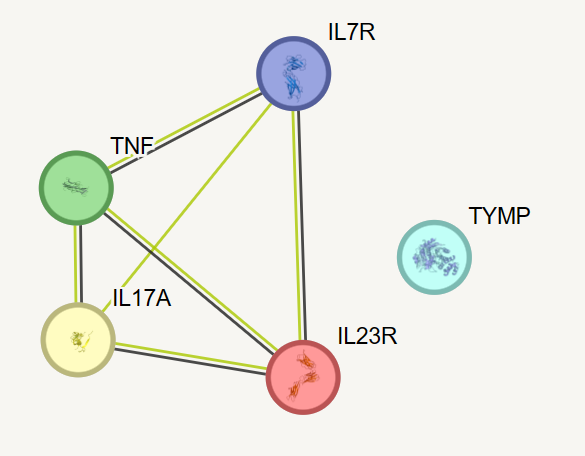

Supplement: Supplementary Figure S1 — Colocalization and Gene Track Plots for Five Proteins. (A, C, E, G, I) display the colocalization plots for IL12B, ERAP1, IL18R1, CCL8, and TNFAIP6, respectively, showing the -log10(P-value) from the GWAS (x-axis) against the -log10(P-value) from the pQTL analysis (y-axis), with colors representing the linkage disequilibrium (LD) r² values. (B, D, F, H, J) present the corresponding gene track plots for these proteins, illustrating the -log10(P-value) along the chromosomal position, indicating genetic variants associated with AS and the candidate proteins. The gene locations and structures are shown below the association signals. [file Image_1.png]

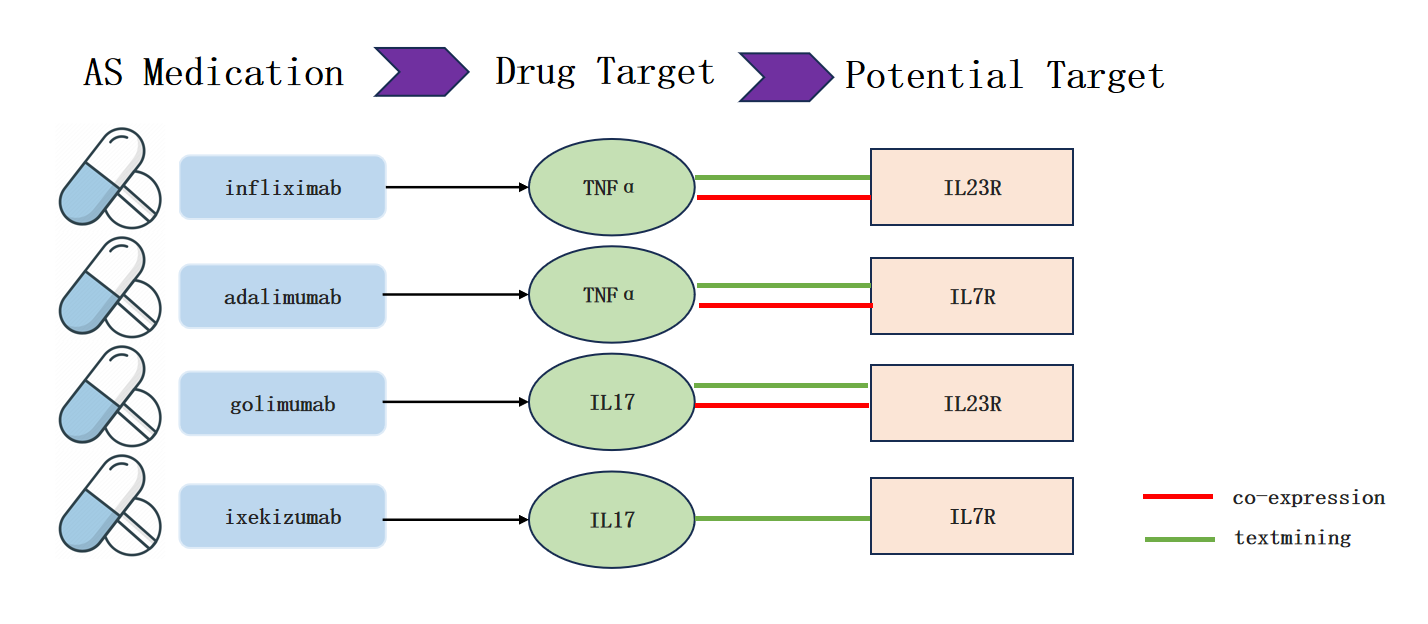

Supplement: Supplementary Figure S2 — Protein-protein interaction networks showing the interactions between three prioritized proteins (IL23R, IL7R, and TYMP) and two current AS drug targets (TNF and IL17). [file Image_2.jpeg]

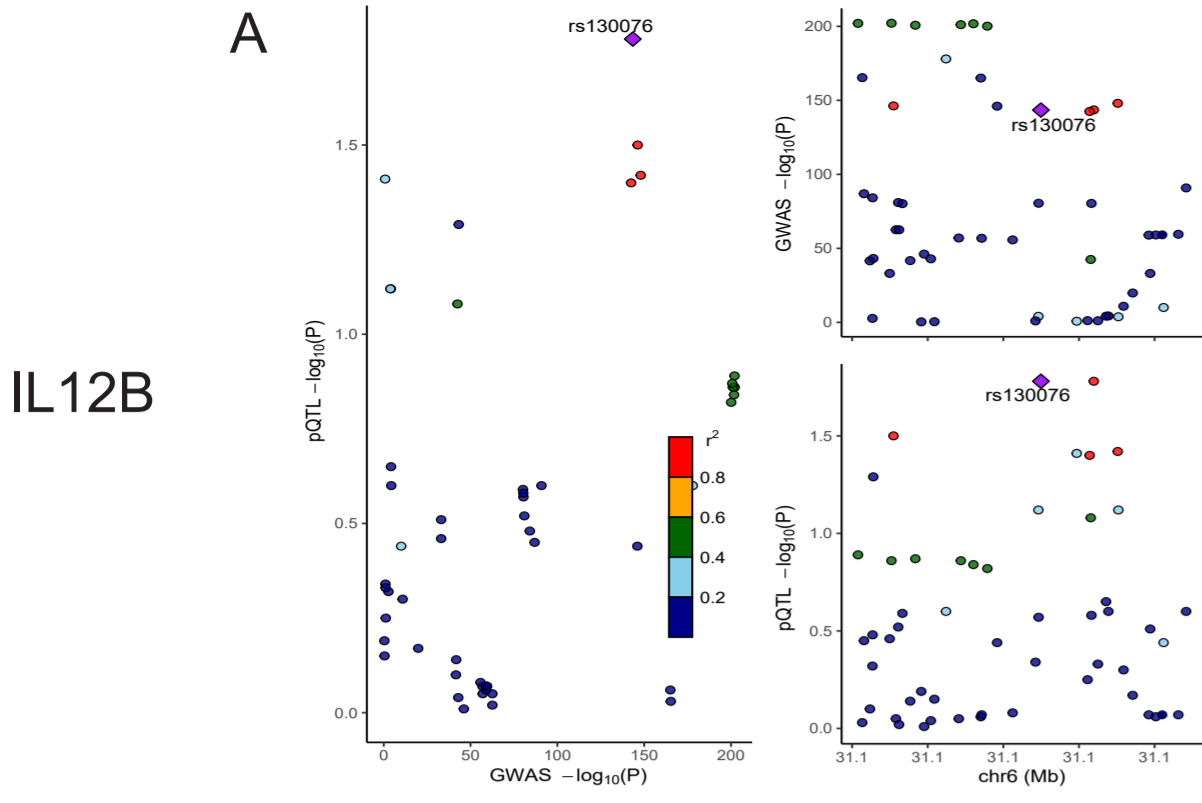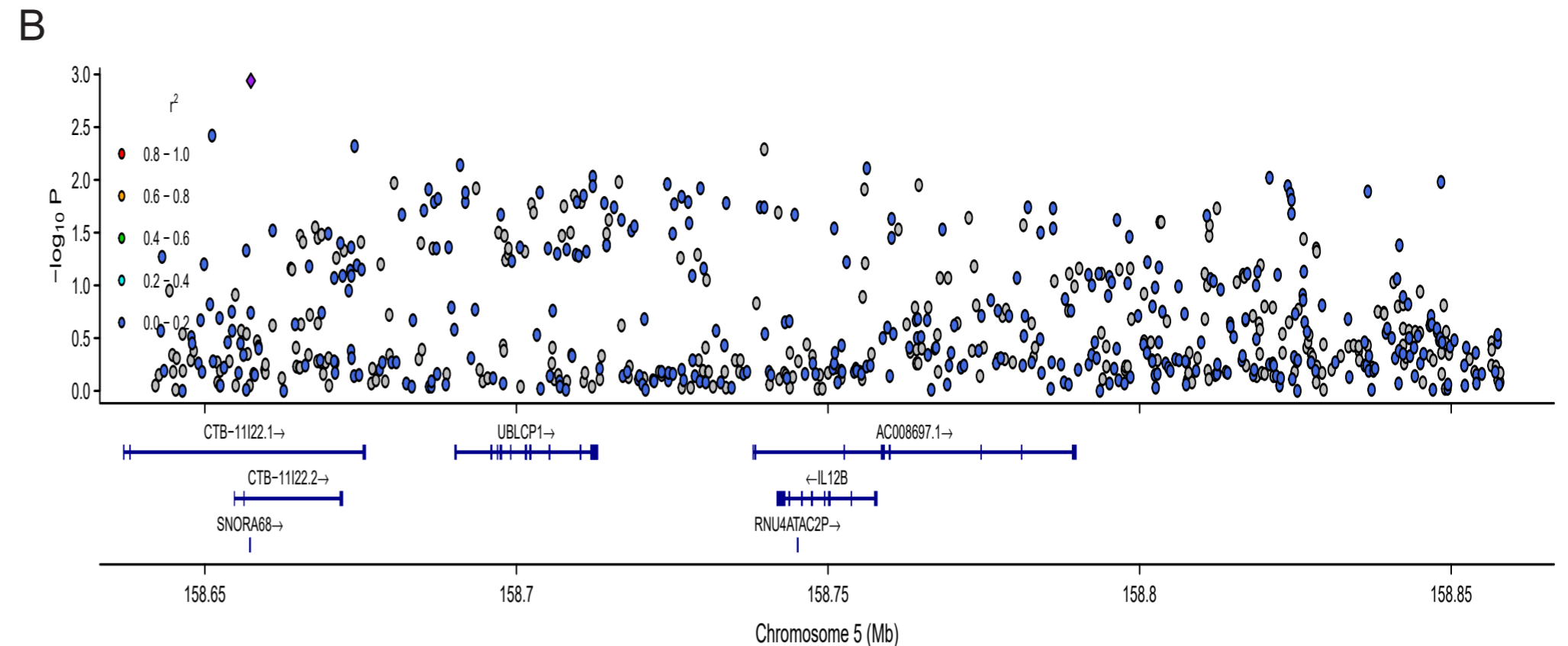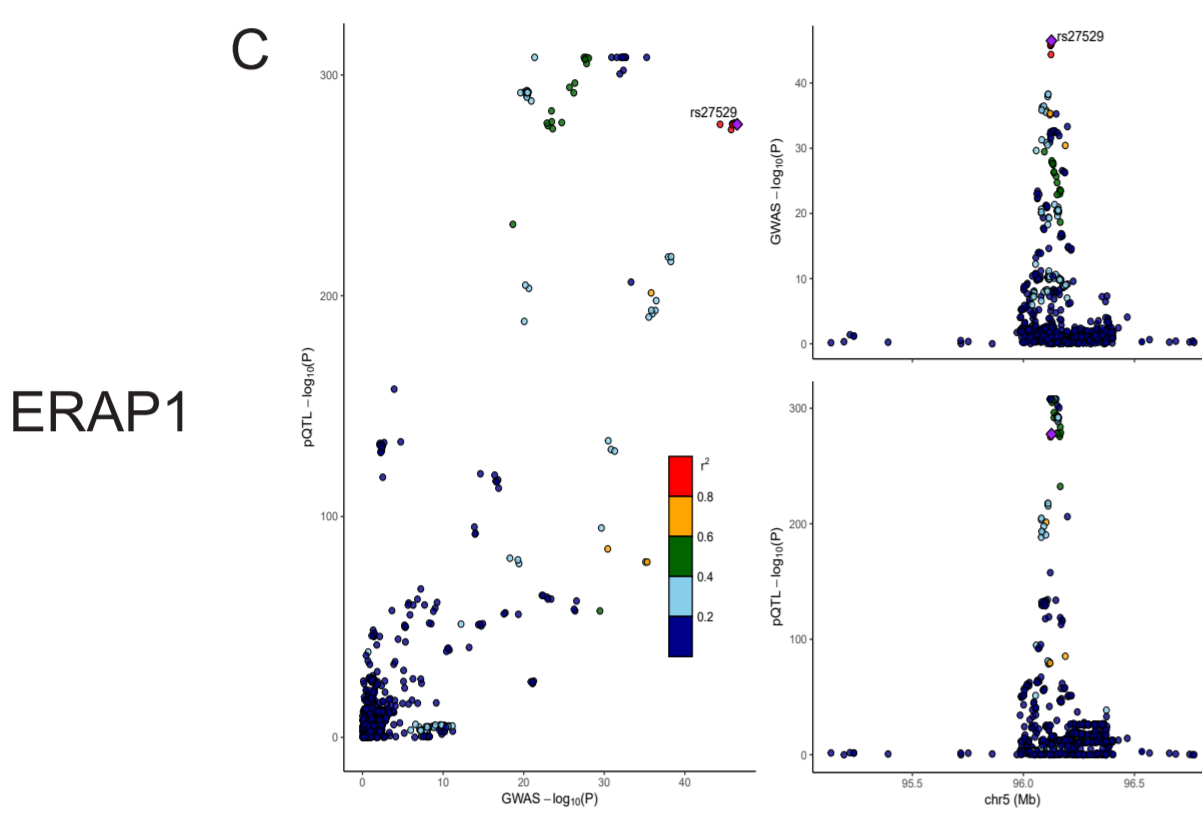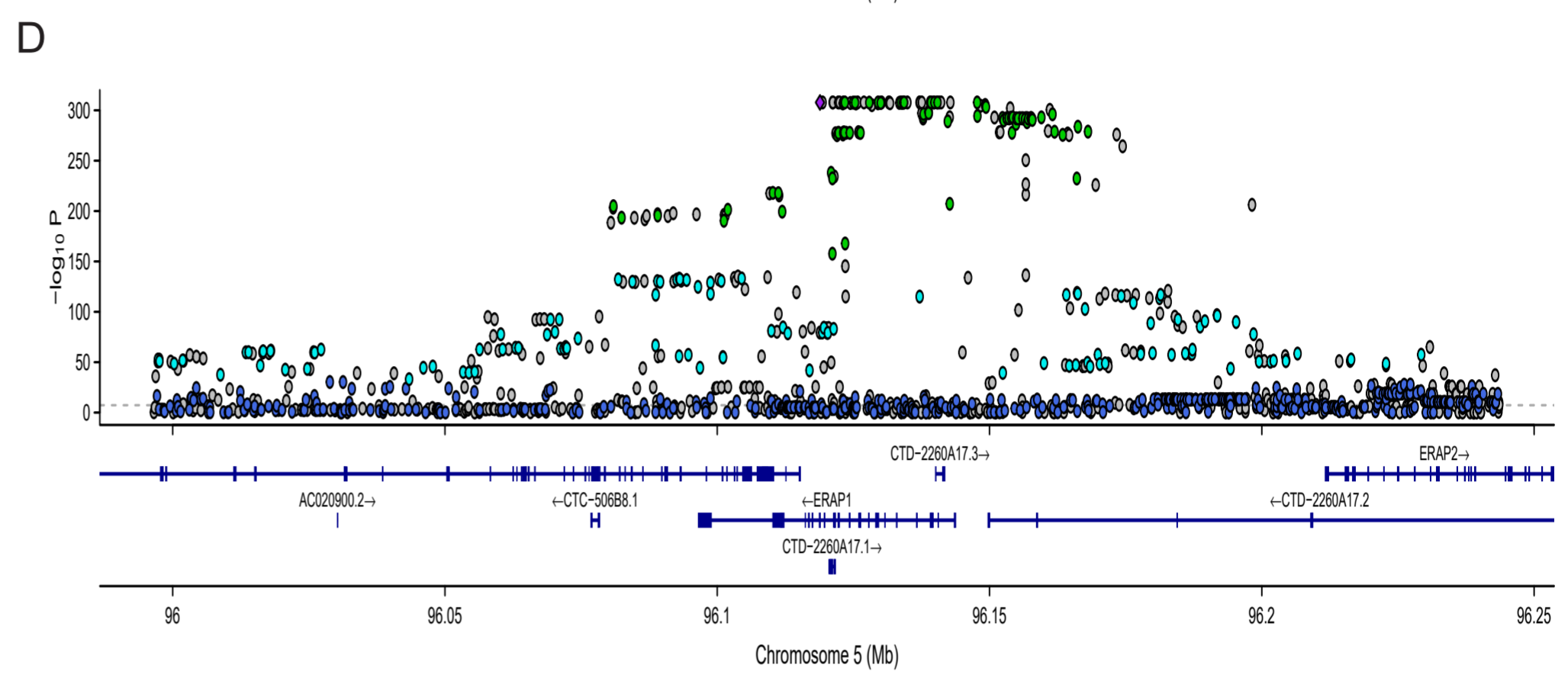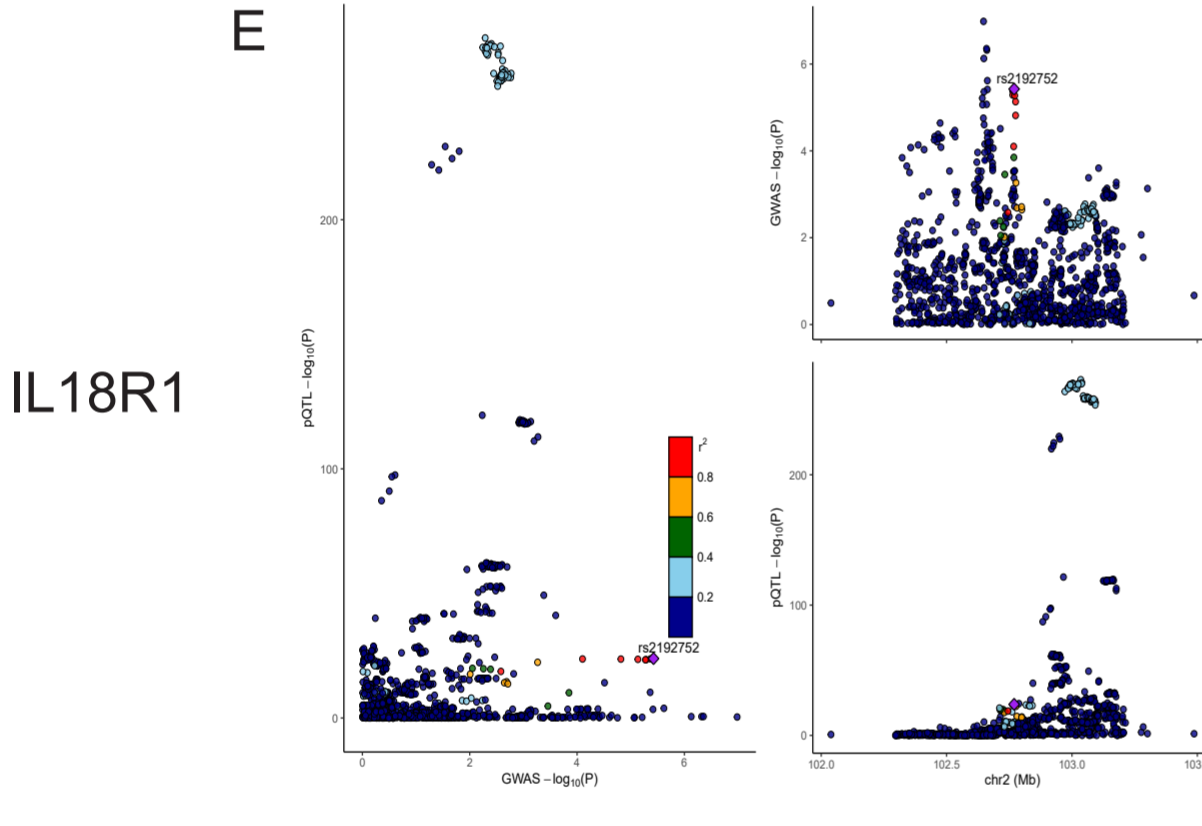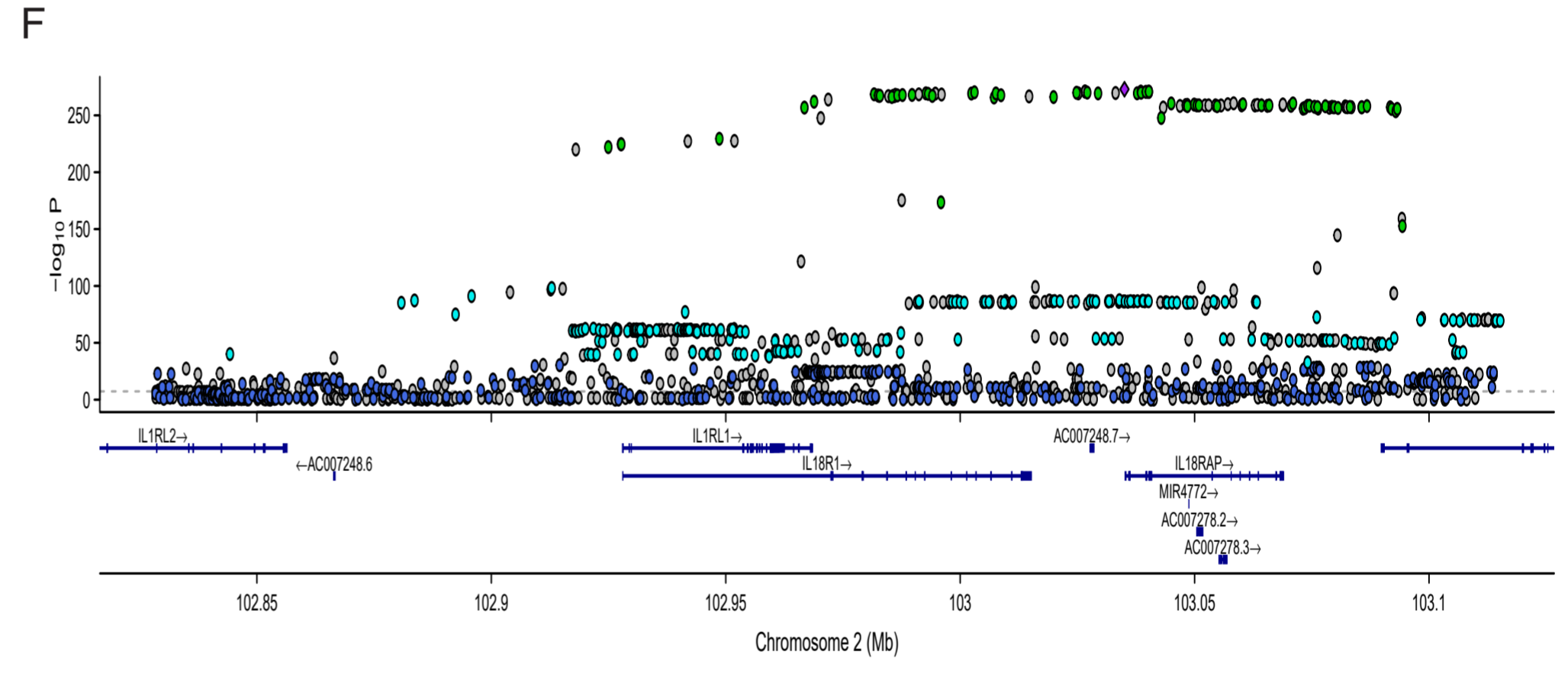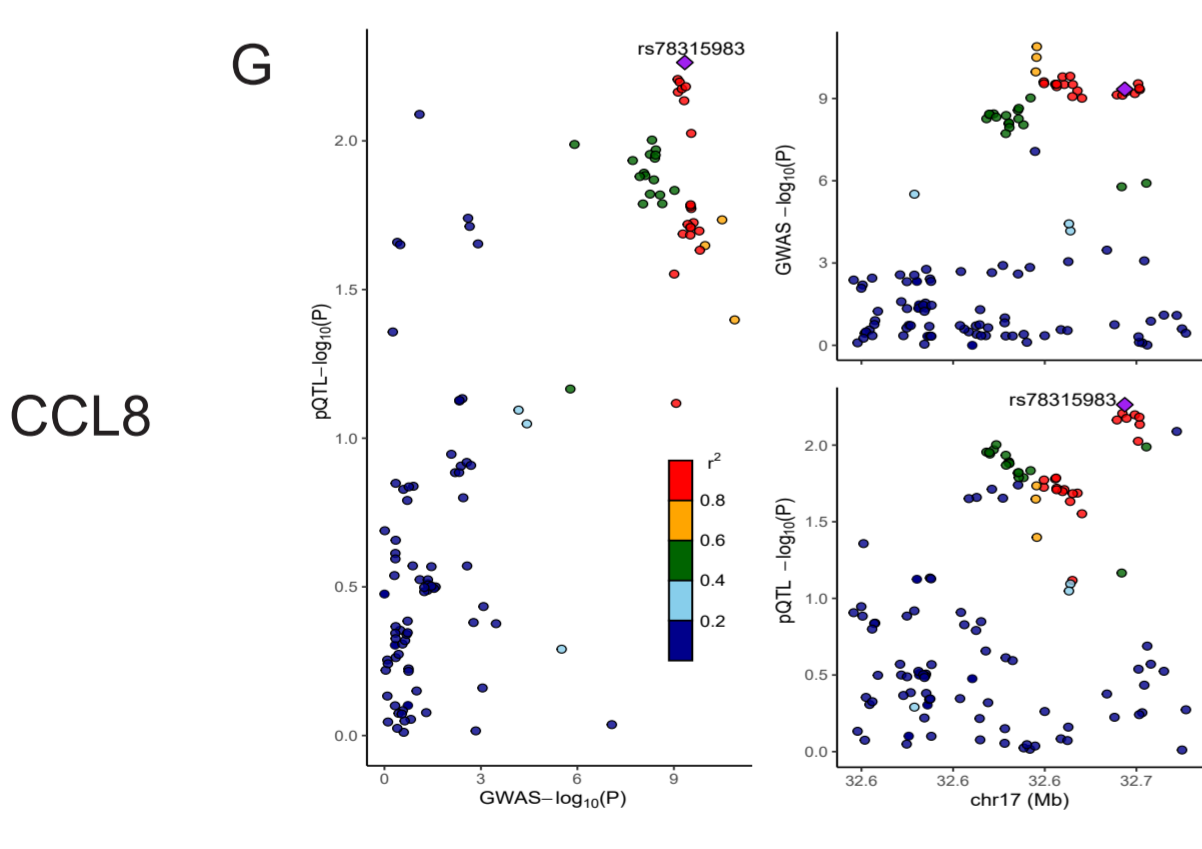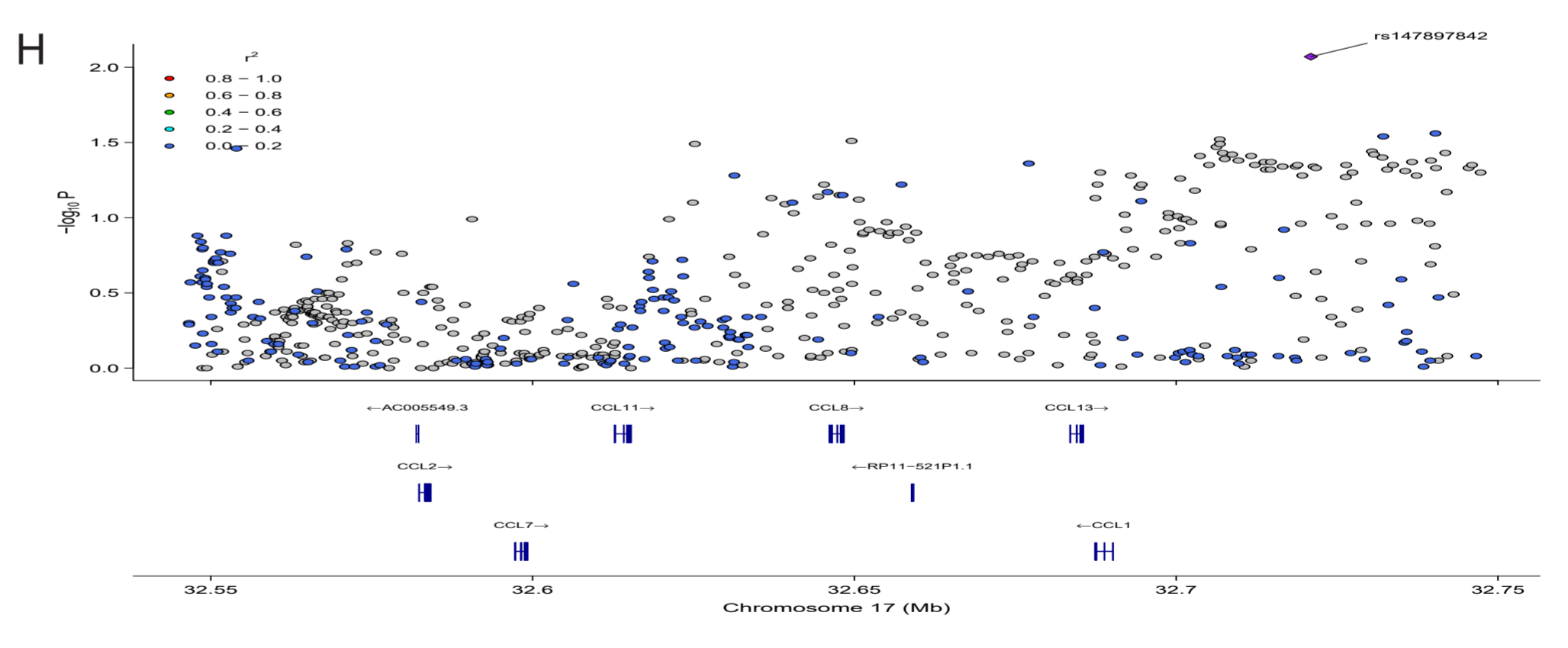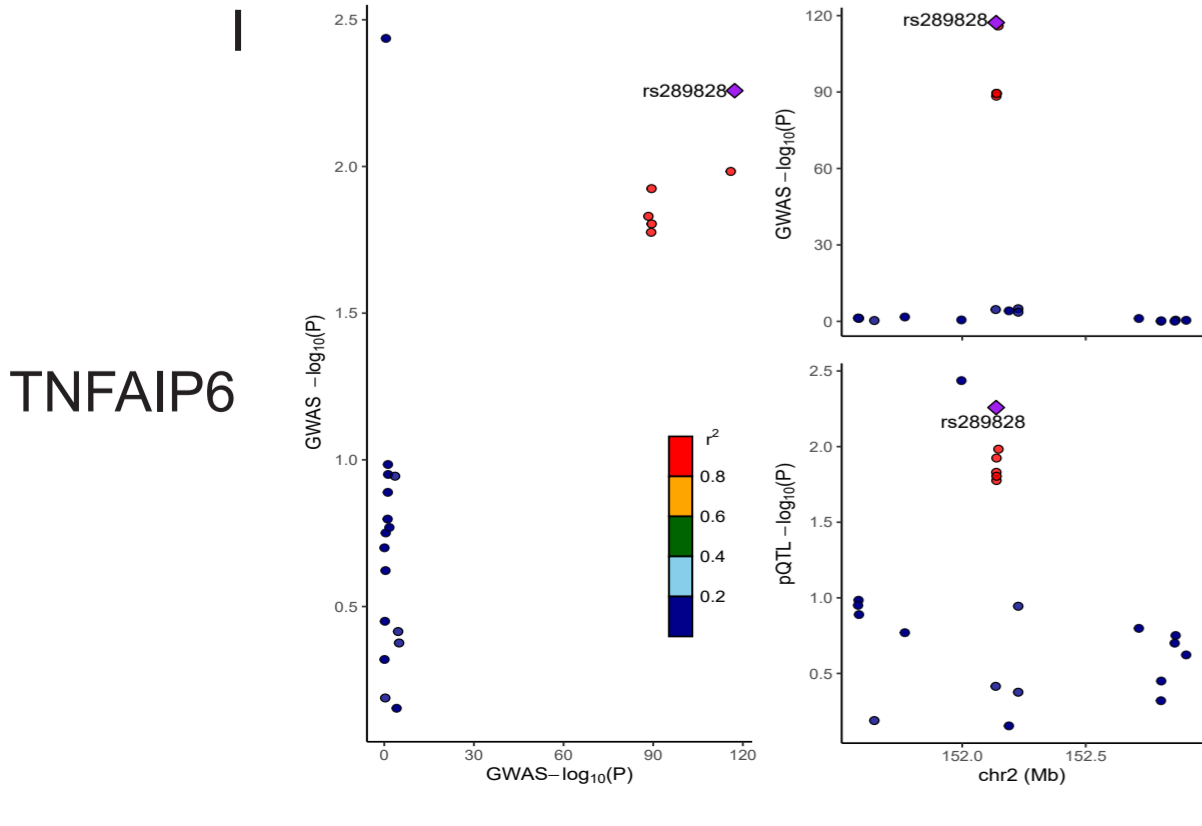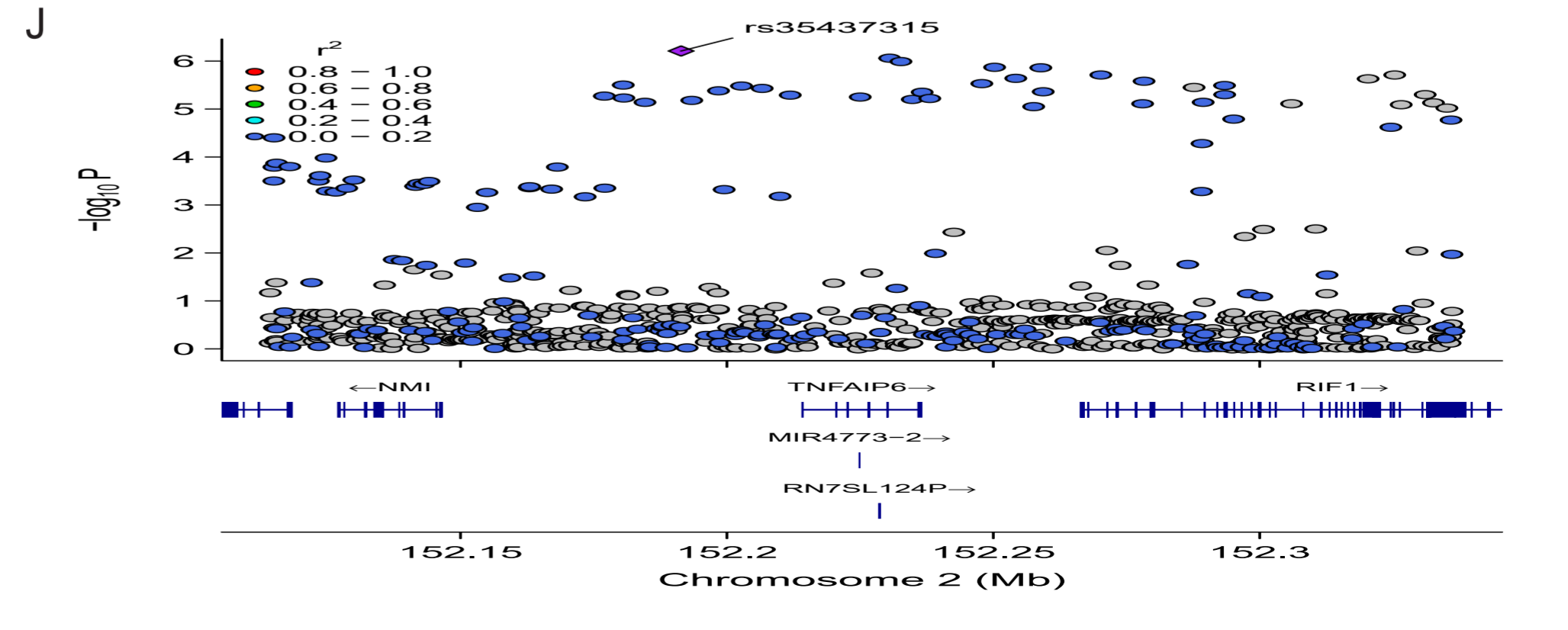

Supplement: Supplementary Figure S3 — Protein-Protein Interaction (PPI) Networks of AS Medications, Drug Targets, and Potential Targets. This diagram illustrates the interactions between current ankylosing spondylitis (AS) medications, their drug targets, and potential new therapeutic targets. AS medications (infliximab, adalimumab, golimumab, and ixekizumab) are shown to target TNF-α and IL17. The potential targets identified are IL23R and IL7R, which are connected to the existing drug targets through co-expression (red lines) and text mining evidence (green lines). [file DataSheet_2.pdf]
