# Supplementary material for: Investigating potential novel therapeutic targets and biomarkers for ankylosing spondylitis using plasma protein screening
Source: Front Immunol. 2024 Aug 9;15:1406041. doi: 10.3389/fimmu.2024.1406041 (PMC11341372; doi:10.3389/fimmu.2024.1406041)
Supplement: Supplementary file 5 [file Table_2.docx]

| Target | Gene | Uniprot ID | Medication | Action | Regulatory Approval | Interaction Score |
| --- | --- | --- | --- | --- | --- | --- |
| Thymidine Phosphorylase | TYMP | P19971 | [TIPIRACIL HYDROCHLORIDE](https://dgidb.org/drugs/rxcui:1670303) | inhibitor | Approved | 3.28 |
| Interleukin 7 Receptor | IL7R | P16871 | [RUXOLITINIB](https://dgidb.org/drugs/rxcui:1193326) | inhibitor | Approved | 3.08 |
| Interleukin 23 receptor | IL23R | Q5VWK5 | CELECOXIB | inhibitor | Approved | 0.45 |

**TableS5. Current medications targeting four potential causal proteins**
