# Supplementary material for: Investigating potential novel therapeutic targets and biomarkers for ankylosing spondylitis using plasma protein screening
Source: Front Immunol. 2024 Aug 9;15:1406041. doi: 10.3389/fimmu.2024.1406041 (PMC11341372; doi:10.3389/fimmu.2024.1406041)
Supplement: Supplementary file 6 [file DataSheet_1.pdf]

STROBE-MR checklist of recommended items to address in reports of Mendelian randomization studies[1 2]

Item No.	Section	Checklist item	Page No.	Relevant text from manuscript
1	**TITLE and ABSTRACT**	Indicate Mendelian randomization (MR) as the study's design in the title and/or the abstract if that is a main purpose of the study	1	"...The genetic profiles of 734 plasma proteins were assessed for causative associations with AS in the International Genetics of Ankylosing Spondylitis Consortium using Mendelian randomization (MR) studies, followed by validation in another cohort. ..."
	**INTRODUCTION**			
2	**Background**	Explain the scientific background and rationale for the reported study. What is the exposure? Is a potential causal relationship between exposure and outcome plausible? Justify why MR is a helpful method to address the study question	2	"...Exploring novel diagnostic biomarkers and therapeutic agents is crucial to provide more effective diagnostic methods and therapeutic options for patients with AS. ...Human proteins are crucial in diverse biological processes and represent the main drug targets. ...MR analyses that integrate GWAS with protein quantitative trait locus (pQTL) data, an approach that could provide important insights for early disease diagnosis and drug target discovery, are lacking in AS research. ..."
3	**Objectives**	State specific objectives clearly, including pre-specified causal hypotheses (if any). State that MR is a method that, under specific assumptions, intends to estimate causal effects	2	"...Genetic instrumental variable analysis involves the use of single nucleotide polymorphisms (SNPs) from genome-wide association studies (GWAS) as the genetic approach to estimate the causal relationship between the exposure and the outcome. ...This study aimed to establish the precise prevention and treatment of AS by discovering potential drug targets from plasma proteins. ..."
	**METHODS**			
4	**Study design and**	Present key elements of the study design early in the article. Consider including a table listing sources of data for all phases of the study. For each data source		

	**data sources**		contributing to the analysis, describe the following:		
		a)	Setting: Describe the study design and the underlying population, if possible. Describe the setting, locations, and relevant dates, including periods of recruitment, exposure, follow-up, and data collection, when available.	3	"...Figure 1 shows the study design. ...The plasma protein data were extracted from plasma proteomics-related publications, whereas the AS data were obtained from the GWAS data of the International Genetics of Ankylosing Spondylitis Consortium (IGAS). ...plasma protein data were obtained from recent publications in Nature Genetics, which integrated data from five previous GWAS datasets, to analyze 738 cis-pQTLs for 734 plasma proteins obtained using the following criteria (Table S1). ..."
		b)	Participants: Give the eligibility criteria, and the sources and methods of selection of participants. Report the sample size, and whether any power or sample size calculations were carried out prior to the main analysis	3	"...We obtained a GWAS summary dataset from the IGAS for the preliminary analysis of AS, which included 9,069 patients and 1,550 controls. ...PQTL data for 4,907 plasma proteins detected among 35,559 participants in the Ferkingstad et al. study and FinnGen GWAS pooled dataset, which included 1,462 patients and 164,682 controls, were used for external validation. ..."
		c)	Describe measurement, quality control and selection of genetic variants	3	... Located outside of the major histocompatibility complex region (chr6, 26–34 Mb). ... exhibiting noteworthy genome-wide associations (p-value < 5*10−8). ...
		d)	For each exposure, outcome, and other relevant variables, describe methods of assessment and diagnostic criteria for diseases	NA	NA
		e)	Provide details of ethics committee approval and participant informed consent, if relevant	NA	NA
5	**Assumptions**		Explicitly state the three core IV assumptions for the main analysis (relevance, independence and exclusion restriction) as well assumptions for any additional or sensitivity analysis	3	"...the instrumental variables (IVs) chosen must meet three essential criteria to ensure valid causal inference: (1) the genetic variants must be directly linked to the exposure; (2) the genetic variants should not be linked to any confounders that could affect the relationship between the exposure and the outcome; (3) the genetic variants should affect the outcome only through the exposure and not via any other pathways. ..."
6	**Statistical methods: main**		Describe statistical methods and statistics used		

	**analysis**				
		a)	Describe how quantitative variables were handled in the analyses (i.e., scale, units, model)	NA	NA
		b)	Describe how genetic variants were handled in the analyses and, if applicable, how their weights were selected	2	"...734 plasma proteins obtained using the following criteria (Table S1). ... Located outside of the major histocompatibility complex region (chr6, 26–34 Mb) . ...This calculation helps to prevent biases due to weak instruments, and an F-statistic above 10 is deemed adequate to overcome such biases. ..."
		c)	Describe the MR estimator (e.g. two-stage least squares, Wald ratio) and related statistics. Detail the included covariates and, in case of two-sample MR, whether the same covariate set was used for adjustment in the two samples	3	"...Five approaches were adopted for estimating the causal effects, including IVW-MR, MR–Egger, weighted median, ...Steiger filtering was also performed to verify the association direction of AS with the plasma proteins. ..."
		d)	Explain how missing data were addressed	NA	NA
		e)	If applicable, indicate how multiple testing was addressed	3	"...We applied the false discovery rate (FDR) correction for multiple testing to select potentially effective causal proteins. ..."
7	**Assessment of assumptions**		Describe any methods or prior knowledge used to assess the assumptions or justify their validity	3	"...an integrative analysis was conducted to identify novel....The "causality" detected through MR could be genetic confounding, or horizontal pleiotropy because of LD. Thus, only cis-pQTL were used as instruments to limit horizontal pleiotropy bias because they directly affect the translation and transcription of the gene of interest. ..."
8	**Sensitivity analyses and additional analyses**		Describe any sensitivity analyses or additional analyses performed (e.g. comparison of effect estimates from different approaches, independent replication, bias analytic techniques, validation of instruments, simulations)	3	"...A sensitivity analysis was performed to verify the accuracy of the data. Specifically, we obtained markedly effective AS genetic instruments from the IGAS dataset. Five approaches were adopted for estimating the causal effects, including IVW-MR, MR–Egger, weighted median, simple mode, and weighted mode. ... Moreover, known diseases and traits sharing SNPs identical to those in our analysis were identified via phenoscanner analysis,... two other genetic instrument sets were used for analysis based on genome-wide significant SNPs and significant variants in the

					validation set. ...”
9	**Software and pre-registration**				
		a)	Name statistical software and package(s), including version and settings used		MR analysis was conducted using “TwoSampleMR” (https://github.com/MRCIEU/TwoSampleMR).
		b)	State whether the study protocol and details were pre-registered (as well as when and where)	NA	NA
	**RESULTS**				
10	**Descriptive data**				
		a)	Report the numbers of individuals at each stage of included studies and reasons for exclusion. Consider use of a flow diagram	NA	NA
		b)	Report summary statistics for phenotypic exposure(s), outcome(s), and other relevant variables (e.g. means, SDs, proportions)	NA	NA
		c)	If the data sources include meta-analyses of previous studies, provide the assessments of heterogeneity across these studies	NA	NA
		d)	For two-sample MR:<br>i. Provide justification of the similarity of the genetic variant-exposure associations between the exposure and outcome samples<br>ii. Provide information on the number of individuals who overlap between the exposure and outcome studies	2-3	“...GWAS summary data for plasma protein levels was obtained from several population-based studies, all of European ancestry. GWAS summary data for Ankylosing spondylitis ...No overlapping participating studies were shared between the GWASs for plasma protein levels and Ankylosing spondylitis. ...”
11	**Main results**				
		a)	Report the associations between genetic variant and exposure, and between genetic variant and outcome, preferably on an interpretable scale	4	After FDR correction, we identified eight significant proteins (Table 1 and Figure 2)
		b)	Report MR estimates of the relationship between exposure and outcome, and the measures of uncertainty from the MR analysis, on an interpretable scale, such as odds ratio or relative risk per SD difference	4	“...including interleukin 7 receptor (IL7R), thymidine phosphorylase (TYMP), interleukin 12B (IL12B), C-C motif chemokine ligand 8 (CCL8), TNF alpha-induced protein 6 (TNFAIP6), ...IL7R (OR = 1.04, 95% CI: 1.01–1.06, P = 7.12e−03), IL12B (OR = 1.08, 95% CI: 1.05–1.11, P = 3.28e−06), ...”

		c)	If relevant, consider translating estimates of relative risk into absolute risk for a meaningful time period	NA	
		d)	Consider plots to visualize results (e.g. forest plot, scatterplot of associations between genetic variants and outcome versus between genetic variants and exposure)	4	Figure 2 and Figure 3
12	**Assessment of assumptions**				
		a)	Report the assessment of the validity of the assumptions	4	"...This calculation helps to prevent biases due to weak instruments, and an F-statistic above 10 is deemed adequate to overcome such biases. ...we used an identical variant and significant variant strategy among diverse datasets to validate the primary finding that eight proteins were associated with AS in the FinnGen cohort. ..."
		b)	Report any additional statistics (e.g., assessments of heterogeneity across genetic variants, such as I^2, Q statistic or E-value)	4	"...heterogeneity was not detected for the analyzed plasma proteins, as shown in Table S2. ..."
13	**Sensitivity analyses and additional analyses**				
		a)	Report any sensitivity analyses to assess the robustness of the main results to violations of the assumptions		"...Following phenotypic scanning, six SNPs from the preliminary analysis were identical to other diseases and traits,...and atopic dermatitis, among others, as detailed in Supplementary Table S3.
		b)	Report results from other sensitivity analyses or additional analyses	4-5	"...External validation of candidate drug targets for AS. Exposures and outcomes from a multicenter dataset were utilized to validate the causal relationship of the abovementioned proteins with AS. ... Moreover, we used an identical variant and significant variant strategy among diverse datasets to validate the primary finding that eight proteins were associated with AS in the FinnGen cohort. We found that ERAP1, IL12B, IL23, IL7R, and IL18R1 proteins are still associated with AS. ...PPI networks demonstrating the interactions of four prioritized proteins..."

		c)	Report any assessment of direction of causal relationship (e.g., bidirectional MR)	4	"...The Steiger filtering method utilizes a statistical test to pinpoint the stronger bidirectional effects. The findings indicated that the eight proteins identified in the primary analysis did not corroborate the presence of reverse causal effects(Table 1). ..."
		d)	When relevant, report and compare with estimates from non-MR analyses	NA	NA
		e)	Consider additional plots to visualize results (e.g., leave-one-out analyses)		Figure 3
	**DISCUSSION**				
14	**Key results**		Summarize key results with reference to study objectives	5	"...ERAP1, IL12B, IL18R1, IL23R, IL7R, and TYMP in plasma proteins showed a causal association with AS risk. It was also found to be associated with AS in the FinnGen cohort using a similar analytical approach, further demonstrating the reliability of the candidate drug targets. ..."
15	**Limitations**		Discuss limitations of the study, taking into account the validity of the IV assumptions, other sources of potential bias, and imprecision. Discuss both direction and magnitude of any potential bias and any efforts to address them	5-6	"...This study had several limitations. First, the primary analyses were performed based on plasma proteomic datasets obtained from several publications, and although most GWAS data sources are aptamer-based, measurement inconsistencies may have caused bias. Second, the preferential proteins contained only one cis-acting SNP but not a trans pQTL... Finally, despite the sensitivity and validation analyses performed in this study, our findings must be interpreted with caution because, although insightful, they are not decisive for causality or as biomarkers of AS...."
16	**Interpretation**				
		a)	Meaning: Give a cautious overall interpretation of results in the context of their limitations and in comparison with other studies	6	"... According to one case–control association study, protective genetic variants are related to a decrease in ERAP1 and ERAP2 function and inhibition of cell surface major histocompatibility complex I expression. ... Mei et al. reported that AS patients ...Previous studies have reported that TNFAIP6 has anti-inflammatory effects in an experimental mouse model of arthritis... the present study suggested that TNFAIP6 may have similar anti-inflammatory protective effects on AS via MR, providing a strong reference for the TNFAIP family

				as the causative genes for AS.
		b) Mechanism: Discuss underlying biological mechanisms that could drive a potential causal relationship between the investigated exposure and the outcome, and whether the gene-environment equivalence assumption is reasonable. Use causal language carefully, clarifying that IV estimates may provide causal effects only under certain assumptions	6	“...TYMP catalyzes reversible thymidine phosphorylation and is suggested to have a critical effect on angiogenesis, tumor growth, migration, and invasion. ... A recent study showed that TNF-α strongly stimulates TYMP expression in fibroblast-like synoviocytes. ...Thus, we hypothesize that TNF-α may affect AS by inducing TYMP expression. ...”
		c) Clinical relevance: Discuss whether the results have clinical or public policy relevance, and to what extent they inform effect sizes of possible interventions	6	“...Our study is the first to perform a comprehensive analysis to investigate the causal relationship between plasma proteins and AS, revealing that circulating IL7R, IL12B, CCL8, IL18R1, IL23R and ERAP1 increase the risk of AS, while TYMP and TNFAIP6 reduce the risk of AS. The proteins identified herein may represent attractive therapeutic targets for AS...”
17	**Generalizability**	Discuss the generalizability of the study results (a) to other populations, (b) across other exposure periods/timings, and (c) across other levels of exposure	6	“... our sample is limited to individuals of European descent, which may restrict the generalizability of ...”
	**OTHER INFORMATION**			
18	**Funding**	Describe sources of funding and the role of funders in the present study and, if applicable, sources of funding for the databases and original study or studies on which the present study is based	6	“...This study was supported by the ...”
19	**Data and data sharing**	Provide the data used to perform all analyses or report where and how the data can be accessed, and reference these sources in the article. Provide the statistical code needed to reproduce the results in the article, or report whether the code is publicly accessible and if so, where	3	“...obtained from recent publications in Nature Genetics, which integrated data from five previous GWAS datasets. ...We obtained a GWAS summary dataset from the IGAS for the preliminary analysis of AS, which included 9,069 patients and 1,550 controls...pQTL data for 4,907 plasma proteins detected among 35,559 participants in the Ferkingstad et al. Study and FinnGen GWAS pooled dataset, which included 1,462 patients and 164,682 controls, were used for external validation. ...”
20	**Conflicts of Interest**	All authors should declare all potential conflicts of interest	6	“... declare that the research was conducted in the absence of any commercial or financial relationships that could be construed as a potential

	conflict of interest. ...”

This checklist is copyrighted by the Equator Network under the Creative Commons Attribution 3.0 Unported (CC BY 3.0) license.

1. Skrivankova VW, Richmond RC, Woolf BAR, Yarmolinsky J, Davies NM, Swanson SA, et al. Strengthening the Reporting of Observational Studies in Epidemiology using Mendelian Randomization (STROBE-MR) Statement. JAMA. 2021;under review.

2. Skrivankova VW, Richmond RC, Woolf BAR, Davies NM, Swanson SA, VanderWeele TJ, et al. Strengthening the Reporting of Observational Studies in Epidemiology using Mendelian Randomisation (STROBE-MR): Explanation and Elaboration. BMJ. 2021;375:n2233.
